# Oxidative stress, hormones, and effects of natural antioxidants on intestinal inflammation in inflammatory bowel disease

**DOI:** 10.3389/fendo.2023.1217165

**Published:** 2023-08-28

**Authors:** Dipak Kumar Sahoo, Romy M. Heilmann, Biswaranjan Paital, Ashish Patel, Virendra Kumar Yadav, David Wong, Albert E. Jergens

**Affiliations:** ^1^ Department of Veterinary Clinical Sciences, College of Veterinary Medicine, Iowa State University, Ames, IA, United States; ^2^ Department for Small Animals, Veterinary Teaching Hospital, College of Veterinary Medicine, University of Leipzig, Leipzig, SN, Germany; ^3^ Redox Regulation Laboratory, Department of Zoology, College of Basic Science and Humanities, Odisha University of Agriculture and Technology, Bhubaneswar, India; ^4^ Department of Life Sciences, Hemchandracharya North Gujarat University, Patan, Gujarat, India

**Keywords:** antioxidants, ulcerative colitis, Crohn’s disease, IBD, oxidative stress, flavonoids, polyphenols, hormones

## Abstract

Inflammatory bowel disease (IBD) is a chronic, relapsing gastrointestinal (GI) disorder characterized by intestinal inflammation. The etiology of IBD is multifactorial and results from a complex interplay between mucosal immunity, environmental factors, and host genetics. Future therapeutics for GI disorders, including IBD, that are driven by oxidative stress require a greater understanding of the cellular and molecular mechanisms mediated by reactive oxygen species (ROS). In the GI tract, oxidative stressors include infections and pro-inflammatory responses, which boost ROS generation by promoting the production of pro-inflammatory cytokines. Nuclear factor kappa B (NF-κB) and nuclear factor erythroid 2–related factor 2 (Nrf2) represent two important signaling pathways in intestinal immune cells that regulate numerous physiological processes, including anti-inflammatory and antioxidant activities. Natural antioxidant compounds exhibit ROS scavenging and increase antioxidant defense capacity to inhibit pro-oxidative enzymes, which may be useful in IBD treatment. In this review, we discuss various polyphenolic substances (such as resveratrol, curcumin, quercetin, green tea flavonoids, caffeic acid phenethyl ester, luteolin, xanthohumol, genistein, alpinetin, proanthocyanidins, anthocyanins, silymarin), phenolic compounds including thymol, alkaloids such as berberine, storage polysaccharides such as tamarind xyloglucan, and other phytochemicals represented by isothiocyanate sulforaphane and food/spices (such as ginger, flaxseed oil), as well as antioxidant hormones like melatonin that target cellular signaling pathways to reduce intestinal inflammation occurring with IBD.

## 1 Introduction

Inflammatory bowel disease (IBD) in humans comprises at least two chronic inflammatory intestinal disorders, defined as Crohn’s disease (CD) and ulcerative colitis (UC). Lesions of CD may occur in the small or large intestine but most commonly involve the colon and rectum as discontinuous areas of transmural inflammation. In contrast, UC affects only the colon and rectum continuously, with inflammation restricted to the mucosa (1). The clinical course of CD is associated with intestinal granulomas, strictures, and fistulae, while these lesions are absent in UC. The underlying mechanism for IBD is believed to result from dysregulated immune responses to environmental factors and the intestinal microbiota in genetically susceptible people (2). These disorders impact millions of people worldwide, with the prevalence of disease in Americans expected to rise by 229% by 2030, relative to the number of diagnoses in 2010 (3).

**Figure 1:**
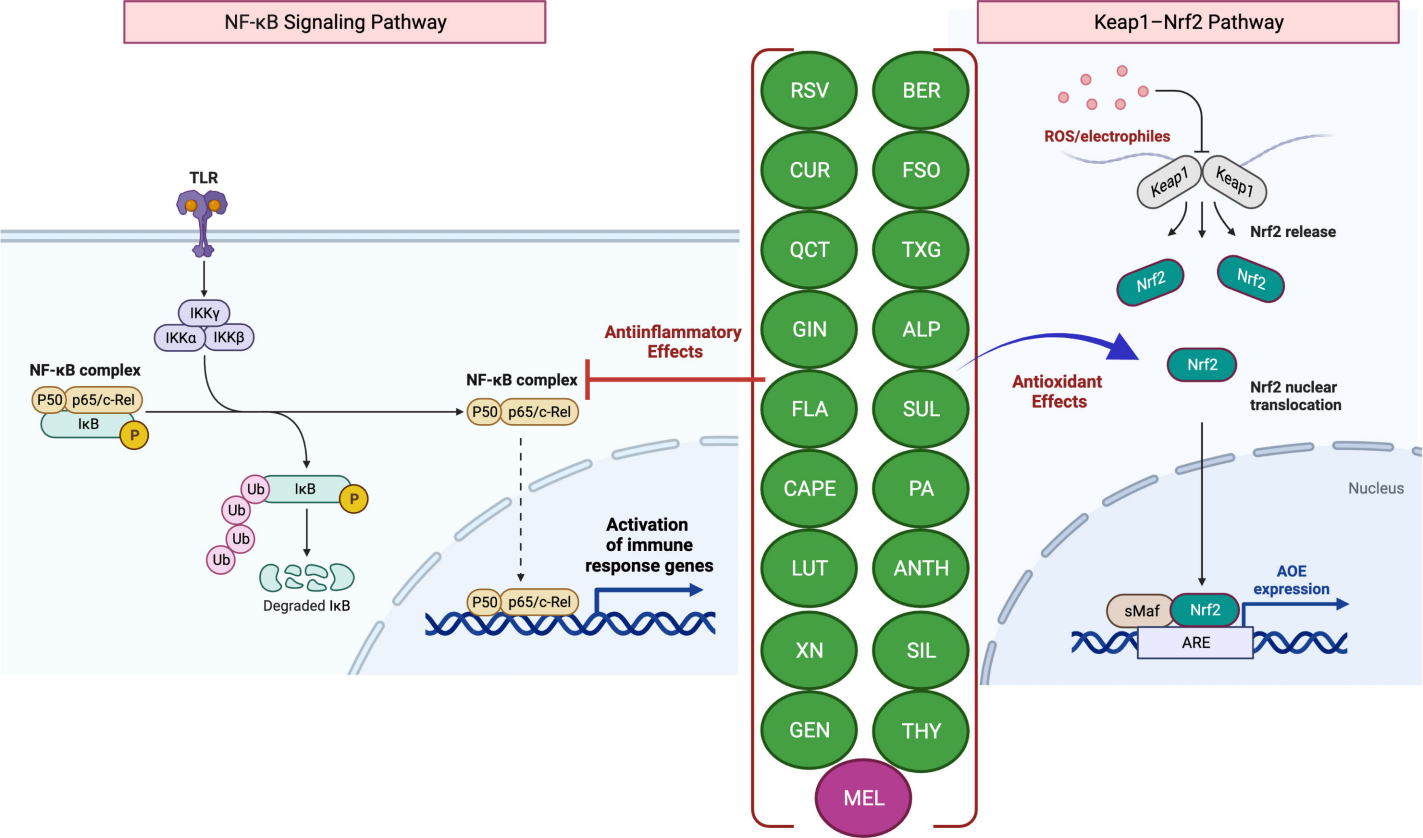
Antioxidant and anti-inflammatory effects of phytochemicals and hormones. Anti-inflammatory mechanisms involve the modulation of nuclear factor-kappa B (NF-κB) pathways, such as the downstream pro-inflammatory effects mediated by Toll-like receptor (TLR) activation. Activation (release of IκB inhibition) and nuclear translocation of NF-κB inhibition, which result in the transcription of several pro-inflammatory genes, can be inhibited by several phytochemicals and hormones (left panel). Kelch-like ECH-associated protein-1 (Keap1)-induced activation and nuclear translocation of nuclear factor erythroid 2-related factor 2 (Nrf2), resulting in an increased expression of antioxidant enzymes (AOEs), can also be inhibited by phytochemicals and hormones (right panel). In the presence of OS, Keap1 relinquishes its binding to Nrf2, thereby enabling the translocation of Nrf2 into the nucleus. Subsequently, Nrf2 forms a complex with small Maf (sMaf) proteins, resulting in the formation of Nrf2/sMaf heterodimer, which then binds to the Antioxidant Response Element (ARE) located on different stress-related gene targets. The figure was produced with BioRender (www.biorender.com; accessed on 17^th^ July 2023). Resveratrol (RSV); Curcumin (CUR); Quercetin (QCT); Ginger (GIN); Flavonoids (FLA); Caffeic acid phenethyl ester (CAPE); Luteolin; (LUT); Xanthohumol (XN); Genistein (GEN); Berberine (BER); Flaxseed oil (α-linolenic acid) (FSO); Sulforaphane (SUL); Tamarind xyloglucan (TXG); Alpinetin (ALP); Proanthocyanidins (PA); Anthocyanins (ANTH); Silymarin (SIL); Thymol (THY); Melatonin (MEL); ubiquitin (Ub); phosphorylation (P); IκB kinase (Iκκ); Mammalian NF-κB family members: NF-κB1 (p50), c-Rel and RelA (p65).

Scientific evidence that increased levels of reactive oxygen species (ROS) but decreased levels of antioxidants contribute to disease pathogenesis establishes a link between ROS and IBD (4, 5). The intestinal mucosa is lined with an epithelial cell monolayer which separates the anaerobic lumen from the highly metabolic lamina propria. Therefore, the intestinal epithelial cells function under a physiological oxygen gradient that is relatively steep (reaching from physiologic hypoxia to physioxia) compared to other cell types. Moreover, during active IBD, there is a significant metabolic shift towards hypoxia seen with mucosal inflammation (pathologic hypoxia). *In vitro* and *in vivo* studies have demonstrated that the activation of the transcription factor hypoxia-inducible factor (HIF) functions as a warning signal in several murine disease models. For example, HIF-1, which is increasingly stabilized in inflammatory lesions, protects against inflammation and IBD by triggering the transcription of several genes that allow the intestinal epithelial cells to operate as an efficient barrier (4). While HIF-1 facilitates adaptive responses to oxidative stress (OS) via nuclear translocation and gene expression regulation, it is well known that mitochondrial HIF-1α protects against OS-induced apoptosis. Several studies have shown that nuclear factor erythroid 2–related factor 2 (Nrf2) helps the anti-inflammatory process by coordinating the recruitment of inflammatory cells and regulating gene expression via the antioxidant response element (ARE) (**Figure 1**) (6). A decrease in the expression of antioxidant/phase II detoxifying enzymes, such as UDP-glucurosyltransferase 1A1, NAD(P)H-quinone reductase-1, heme-oxygenase-1, and glutathione S-transferase Mu-1 was linked to the increased severity of colitis in Nrf2-deficient mice (7), while *Nrf2* overexpression was reported to improve UC (8).

## 2 Concepts of cellular and ROS damage

The intestinal mucosa in people with IBD (e.g., CD) is typically infiltrated with numerous inflammatory cells, including neutrophils, macrophages, and lymphocytes (9, 10). Uncontrolled immune responses are driven by the excessive activity of effector T-lymphocytes and their increased production of pro-inflammatory cytokines (e.g., tumor necrosis factor-alpha (TNF-α), interleukin (IL)-1β, and IL-6) and chemokines, which, along with other inflammatory mediators, cause tissue damage and perpetuate the inflammatory response (11). Typically, the equilibrium between proinflammatory cytokines (such as TNF-α, IL-1, IL-6, IL-8, IL-17, and IL-23) and anti-inflammatory cytokines, such as IL-5, IL-10, IL-11, and transforming growth factor β (TGF-β) is closely regulated within the GI mucosa (12). The pathogenesis of IBD is characterized by an imbalance between T helper (Th) cells and regulatory T cells, specifically the impaired tolerance of regulatory T cells. While CD is distinguished by inflammation mediated by Th1 cells, which results in the overproduction of IL-12, IL-17, and IL-23, UC is marked by cytokines such as IL-4, IL-5, IL-10, and IL-13, which are produced by Th2-type T cells (13).

The active inflammatory process is coupled with the generation and release of ROS from infiltrating immune cells. Principal ROS produced by inflammatory cells include superoxide (O_2_^•–^), hydroxyl radical (·OH), hydroperoxyl radical (HO_2_·), nitric oxide (NO), and singlet oxygen (^1^O_2_) (14). Furthermore, ROS upregulates genes involved in innate and adaptive immune responses to amplify mucosal inflammation (12). ROS and other inflammatory markers released in the inflamed mucosal environment cause progressive cellular and molecular damage, resulting in increased tissue destruction. The most common cellular targets for ROS include cell membrane lipids, proteins, and DNA which causes lipid peroxidation (LPx), enzymatic dysfunction, and DNA damage, respectively (15–17). OS in IBD occurs due to an imbalance between oxidant and antioxidant substances in affected tissues (18). This review also evaluates the role of antioxidants and hormones in the crosstalk between OS and inflammation in IBD.

## 3 Clinical studies in companion animals

Canine chronic inflammatory enteropathies (CIE) refer to a group of intestinal disorders characterized by persistent or recurrent gastrointestinal (GI) signs and variable intestinal inflammation (19, 20). The prevalence of CIE in referral veterinary practice is estimated at 2%, and it is generally subclassified by the response to different therapeutic trials (20). The different disease phenotypes include food-responsive enteropathy (FRE), antibiotic-responsive enteropathy (AE), steroid-responsive enteropathy (SRE), often termed idiopathic IBD, and nonresponsive enteropathy (NRE) (20–22). While the cause of canine CIE is unknown, it is also recognized as a multifactorial disorder resulting from a complex interplay among the environment (e.g., diet, microbiome), mucosal immunity, and host genetics that initiates and drives chronic intestinal inflammation, like human IBD (19).

There are few clinical studies evaluating the role of OS in dogs with CIE. In one case-control study, colonic lavage analytes as markers of mucosal inflammation were compared between healthy dogs and dogs with biopsy-confirmed idiopathic IBD (23). Polyethylene glycol solution was administered into the colon via rectal balloon catheter prior to colonoscopy and was then analyzed for total protein, IgG, and nitrite concentrations and myeloperoxidase (MPO) activity. Results showed that mean nitrite and IgG concentrations were higher in lavage samples from idiopathic IBD dogs compared with samples from healthy dogs. Serum metabolite profiles have also demonstrated a potential relevance of OS in the pathogenesis of dogs affected by idiopathic IBD (24). Using an untargeted metabolomic approach, gluconic acid lactone and hexuronic acid increased in the serum of idiopathic IBD dogs when compared to samples from healthy dogs. Gluconic acid is an oxidized derivative of glucose that can scavenge free radicals, and hexuronic acid is a biologically active form of vitamin C that functions as an antioxidant by donating electrons. Interestingly, there were no significant changes in serum metabolite profiles in dogs with idiopathic IBD following medical therapy, despite clinical improvement.

Several other studies have investigated serum biomarkers of OS in dogs with CIE at diagnosis and in response to medical treatment. In one study, trolox equivalent antioxidant capacity (TEAC), cupric reducing antioxidant capacity (CUPRAC), ferric reducing ability of the plasma (FRAP), total thiol concentrations, and paraoxonase-1 (PON1) activity were evaluated in serum to determine the antioxidant response in dogs with idiopathic IBD. Additionally, ferrous oxidation-xylenol orange (FOX), thiobarbituric acid reactive substances (TBARS), and ROS concentrations in serum were determined (25). The mean concentrations of all antioxidant biomarkers except FRAP were lower, and the oxidant markers were higher in the sera of dogs with idiopathic IBD than in healthy controls. Another study showed lower serum fatty acid concentrations in dogs with CIE than in healthy dogs, indicating dysregulation of both pro-inflammatory (arachidonic acid and cyclooxygenase pathways) and anti-inflammatory (omega-3 essential fatty acids) mediators (26). Perturbations in these mediators in the face of chronic intestinal inflammation are a recognized feature of IBD in people (27).

Differences in systemic phospholipids were reported in another study involving dogs with idiopathic IBD and FRE (28). Overall, disease severity and treatment (e.g., elimination diet alone for FRE versus elimination diet and immunosuppressive dose of prednisolone for idiopathic IBD) were the most significant variables affecting phospholipid profiles at diagnosis. After treatment, a shift of phospholipid species from phosphatidylcholine to lysophosphatidylcholine was observed for both disease groups, presumably caused by an increase in anti-inflammatory lipid mediators (lipoxins and resolvins). The effects of dietary supplements and diet therapy on metabolomic changes in dogs with CIE have been investigated in other treatment trials. In one controlled trial, dogs with idiopathic IBD were randomized to treatment with either a hydrolyzed diet alone or a hydrolyzed diet supplemented with prebiotics (PRE) and glycosaminoglycans (GAG) (29). Results indicated that the majority of metabolomic changes involved several different lipid classes (glycerophospholipids, sphingolipids, and di- and triglycerides) and that both treatments increased beneficial metabolites in serum lipid profiles. In addition, co-treatment with PRE + GAG was associated with the greatest increase in lipid metabolites suggesting a possible additional beneficial effect in dogs with idiopathic IBD. Another randomized controlled trial in dogs with idiopathic IBD involved combination therapy with hydrolyzed diet and oral chondroitin sulfate + PRE versus hydrolyzed diet alone (30). The supplement group showed decreased serum levels of paraoxonase-1 after 60 days of treatment, whereas the placebo group showed reduced serum total antioxidant capacity after 120 days. A decrease in the intestinal histologic score was observed only in the supplement group post-treatment. Additionally, breed-specific changes in the fecal metabolomic profile have been reported in Yorkshire Terriers, which show an increased susceptibility to CIE and protein-losing enteropathy. Here, changes in bile acid, fatty acid, and sterol metabolism that only partially recovered with successful treatment were observed (31).

While most studies have investigated the pathomechanisms of OS in chronic gastroenteritis, other studies have evaluated the role of antioxidants in acute enteropathy and in mitigating OS induced by surgery. In one study, a comparison of the OS status of dogs with uncomplicated acute diarrhea (AD) was compared to healthy controls (32). Both cohorts were screened for clinical and laboratory abnormalities as well as routine redox indices (reactive oxygen metabolites [dROMs], serum antioxidant capacity [SAC], and the oxidative stress index [OSi]). Dogs with AD showed increased levels of dROMs and OSi values (calculated as the ratio between dROMs and SAC) as compared to control indices.

Summarizing, different metabolomic studies in dogs with CIE also show disturbances in serum and fecal metabolites reflective of OS at diagnosis. Notably, disturbances in lipid metabolism appear to be a common denominator across multiple studies. Moreover, treatment using a hydrolyzed diet with or without different dietary supplements improves several different measures of OS in most animals showing clinical remission. Given the importance of these metabolites in mediating chronic intestinal inflammation, additional well-designed and sufficiently powered clinical trials in dogs with CIE are warranted.

## 4 Oxidative stress and antioxidant defenses

The most prevalent antioxidant signaling pathway is the Kelch-like epichlorohydrin-related protein-1 (Keap1)/Nrf2-ARE signaling pathway (33, 34). As an inactive compound with its cytosolic repressor, Keap1, Nrf2 is sequestered in the cytoplasm. In response to OS that oxidizes two SH groups, Nrf2 is dissociated from the inhibitory protein Keap1 and translocated to the nucleus, where it binds ARE to activate the transcription of antioxidant genes (33, 34) (**Figure 1**).

**Figure 2:**
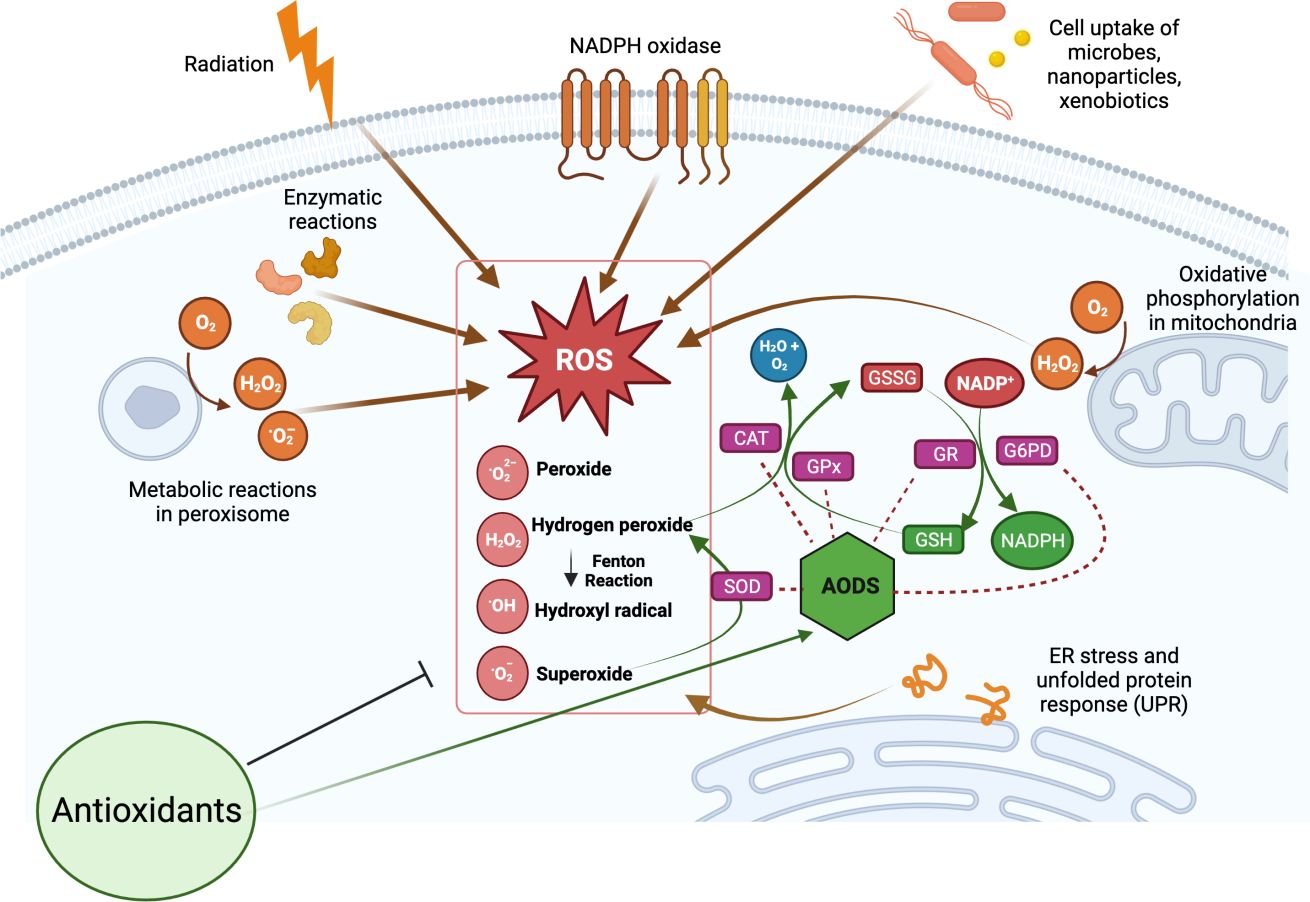
Overview of cellular reactive oxygen species (ROS) generation and their scavenging by antioxidant defense system (AODS). Supplemental antioxidants can help lower oxidative stress by scavenging free radicals, blocking enzymes that produce ROS, or stimulating AODS enzymes and molecules. The figure was produced with BioRender (www.biorender.com; accessed on 17^th^ July 2023). Superoxide dismutase (SOD); catalase (CAT); glutathione peroxidases (GPx); glutathione reductase (GR); reduced glutathione (GSH); oxidized glutathione (GSSG); glucose-6-phosphate dehydrogenase (G6PD).

The production of ROS is a natural consequence of biological metabolism (**Figure 2**). The beneficial effects of ROS are seen in a variety of physiological processes at low and moderate concentrations, including the killing of invading pathogens, the healing of wounds, and the repair of damaged tissues. Aerobic organisms possess a wide range of antioxidants that are critical to their survival. Antioxidants can be classified as either enzymatic or non-enzymatic, depending on their functions. While the antioxidant defense enzyme superoxide dismutase (SOD) converts the superoxide anion to hydrogen peroxide, catalase (CAT), peroxiredoxins (Prxs), and glutathione peroxidases (GPx) are examples of antioxidant enzymes (AOEs) that catalyze the breakdown of hydrogen peroxide (35, 36). Recent studies investigating refined kinetic measurements show that Prxs remove more than 90% of cellular peroxides in comparison to other antioxidant defense enzymes like CAT and GPx (35). Copper and iron ion-sequestering molecules, heme oxygenase, lipoic acid, uric acid, coenzyme Q, and bilirubin are all examples of non-enzymatic antioxidants present *in vivo* (37). In the non-enzymatic antioxidant defense system, glutathione (GSH) plays a crucial function as the most abundant cytosolic thiol. Glutathione can protect cells from free radicals and pro-oxidant damage because it also serves as a cofactor for other antioxidant and detoxifying enzymes, including GPx, glutathione S-transferases (GST), and glyoxalases. Thioredoxin (Trx) serves as a co-substrate molecule for Prxs, and its reducing capabilities are essential to the antioxidative activities of Prxs. Trx and GSH need glutathione reductase (GR) and thioredoxin reductase (TR), in addition to NADPH, to retain their reducing capabilities (38). During the process of GST-mediated detoxification of electrophilic compounds and xenobiotics, GSH functions as a cofactor (39). Following detoxifying interactions of vitamin E with lipid peroxyl radicals (LOO·), GSH can replenish the vitamin E pool (40). Antioxidants that can regenerate their original qualities through interactions with other antioxidants are referred to as an “antioxidant network” (41). Growing research suggests that pathological states characterized by elevated ROS levels are associated with diminished enzymatic and non-enzymatic antioxidant activity (34, 39, 42–59). The signaling pathways of nuclear factor kappa B (NF-κB), mitogen-activated protein kinase (MAPK), and signal transducer and activator of transcription 3 (STAT3) are among the major targets that can be influenced by ROS. Therefore, these pathways play a crucial role in defending against the effects of OS and can be used to identify antioxidant food ingredients or to develop therapeutics for diseases such as IBD. Myeloperoxidase is frequently overexpressed in a variety of inflammatory disorders (60, 61), including chronic gastroenteritis. As a lysosomal enzyme, MPO is secreted into the phagosome of neutrophils after degranulation, where it catalyzes the formation of strong oxidants, such as hypohalous acid (HOX; X = Cl or Br) with potent antibacterial properties. When generated at an improper location, time, or concentration, HOX can potentially damage host tissue. MPO-mediated damage is associated with a number of disorders in people, including IBD (60, 62). High leukocyte infiltration in the inflamed mucosa generates high levels of ROS, which triggers OS and causes cellular and tissue damage seen with inflammation (63).

## 5 ROS generation in the gastrointestinal tract

The GI tract is one of the primary sources of ROS. Although epithelium acts as a physical and antimicrobial barrier, ingested materials and enteric pathogens can promote inflammation by stimulating the production of proinflammatory cytokines, which further contribute to OS. Oxidative stress is a contributing factor in the development of several GI pathological disorders, such as gastroduodenal ulcers, GI cancer, and IBD. Acute and chronic GI disorders in humans and animal models are characterized by increased ROS production or decreased counteracting antioxidant mechanisms, both of which disrupt redox homeostasis (12, 32, 64, 65).

Oxidative stress-induced damage in chronic intestinal disorders is associated with mucosal infiltration by activated leukocytes, which produce excessive ROS that overwhelm the tissue’s antioxidant defenses and perpetuate or exacerbate mucosal inflammation. Several ROS generated by unstable types of oxygen, including the superoxide ion, hydrogen peroxide, and hydroxyl radicals, are the principal pro-oxidant molecules (12).

The intestinal epithelium has been acknowledged as a crucial factor in the development of IBD due to its dual nature of exhibiting both immune and organ-specific functions. In the context of mucosal inflammation, the activation of NADPH oxidase (NOX) and inducible nitric oxide synthase (iNOS) by inflammatory cytokines leads to the production of superoxide and nitric oxide by intestinal epithelial cells (IECs), neutrophils, and macrophages (12). IECs generate an increased amount of ROS/RNS through the activation of NOX and iNOS. The presence of excessive ROS has the potential to cause harm to cytoskeleton proteins, which may modify tight junctions to increase intestinal permeability. Ultimately, this disrupted intestinal epithelial barrier leads to further mucosal inflammation (66). Thus, the initiation of IBD can be attributed to inflammation of the GI tract caused by OS. The microvascular network encircling the epithelial cells can attract inflammatory mediators causing more tissue damage and an escalation of intestinal inflammation. Morphologic lesions associated with intestinal inflammation include goblet cell depletion, decreased production of mucous, development of ulcers and/or hyperplasia of colonic crypt cells (12, 67).

Both ROS and RNS have been implicated in IBD pathogenesis, with a particular role in CD initiation and progression (68). With inflammation, the production of ROS by leukocytes and monocytes increases along with prostaglandins and leukotrienes (e.g., eicosanoids derived from arachidonic acid metabolism) (69, 70). ROS in the GI tract is produced by infiltrating neutrophils and macrophages, as well as IECs. Elevated blood levels of 8-hydroxy-2′-deoxyguanosine (8-OHdG) may serve as a biomarker for OS in people with IBD (13). In murine colitis models, systemic depletion of macrophages or neutrophils results in decreased ROS/RNS production, reduced concentrations of proinflammatory cytokines, and mitigation of intestinal inflammation (71).

These ROS promote cell damage and harm tissue integrity by preventing the accumulation of antioxidant defenses in host cells. For example, oxidative damage is observed in the intestinal tissues and peripheral blood leukocytes of patients with CD (72). Moreover, CD patients have lower levels of antioxidant vitamins A, C, E, and beta-carotene in their blood and intestinal mucosa, as well as reduced activity of key cellular AOEs such as glutathione peroxidase and SOD (73). Oxidative stress and redox signaling pathways, especially that involving NF-ĸB, are also involved in active IBD (**Figure 1**). Since the redox status of mucosal glutathione is associated with inflammation and disease progression, impaired mucosal antioxidant defenses likely contribute to the development of UC (74).

**Figure 3:**
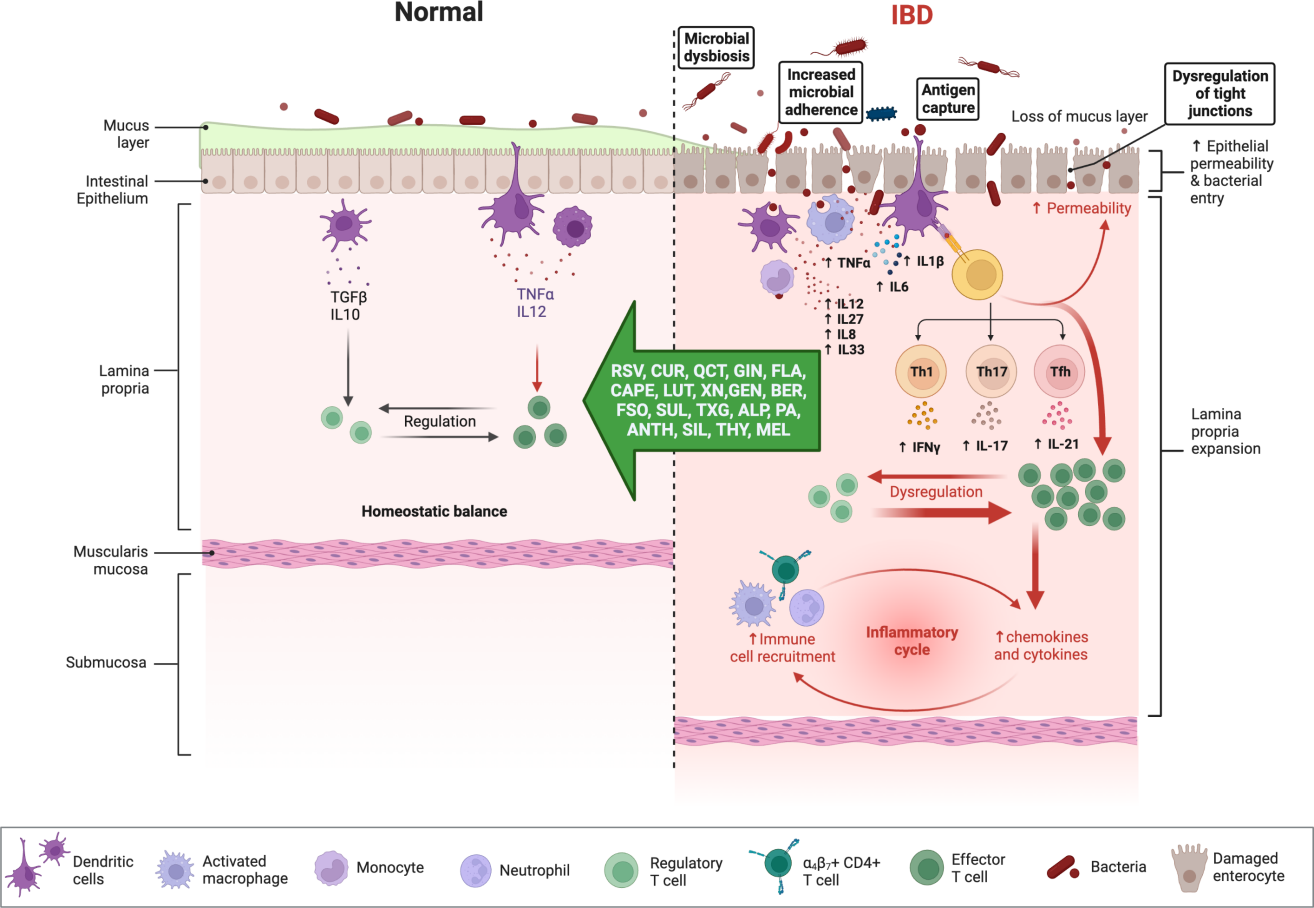
Immune responses in IBD and anti-inflammatory effects of phytochemicals and hormones. Whereas the physiologic state of the gastrointestinal (GI) immune system is dominated by immune tolerance, which maintains homeostatic balance, disturbances with IBD are associated with an exaggerated (i.e., pro-inflammatory) immune response, intestinal dysbiosis, and compromised intestinal barrier function. Pro-inflammatory mediators can perpetuate and exacerbate these dysregulated immune responses, while several phytochemicals and hormones can shift this imbalance toward homeostasis. The figure was produced with BioRender (www.biorender.com; accessed on 17^th^ July 2023). Resveratrol (RSV); Curcumin (CUR); Quercetin (QCT); Ginger (GIN); Flavonoids (FLA); Caffeic acid phenethyl ester (CAPE); Luteolin; (LUT); Xanthohumol (XN); Genistein (GEN); Berberine (BER); Flaxseed oil (α-linolenic acid) (FSO); Sulforaphane (SUL); Tamarind xyloglucan (TXG); Alpinetin (ALP); Proanthocyanidins (PA); Anthocyanins (ANTH); Silymarin (SIL); Thymol (THY); Melatonin (MEL); interferon-gamma (IFNγ); tumor necrosis factor-alpha (TNF-α); transforming growth factor β (TGF-β); interleukin (IL). Naive CD4 T cells differentiated Th1, Th17, Tfh (follicular T helper), and Treg (T regulatory) subsets.

Chronic NF-ĸB stimulation promotes cellular infiltration and mucosal inflammation by increasing the transcription of proinflammatory cytokines (e.g., IL-6, IL-8, IL-16, and TNF-α) and by degrading the intestinal barrier through increased apoptosis of intestinal epithelial cells (**Figure 3**) (75), up-regulation of metalloproteinases which digest mucosal cells, and the release of ROS metabolites that activate NF-ĸB to further impair barrier stability (76). Matrix metalloproteinases (MMPs), a disintegrin and metalloproteinase (ADAMs), and tissue inhibitors of metalloproteinases (TIMPs) are involved in the regulation of the inflammatory response (77). The intestinal mucosa of IBD patients demonstrates an up-regulation of MMPs and ADAM17 (TNF-α converting enzyme; TACE), which is commonly correlated with disease severity but is not accompanied by an up-regulation of TIMP (78). It seems possible that the expression of different MMPs in IBD is affected by the imbalance between oxidants and antioxidants, given the importance of OS in the etiology of the disease (79). In addition to normalizing the intracellular redox state, antioxidants directly influence the regulation of MAPK and transcription factors and can reduce the production of MMPs, restoring their levels to normal (79).

One of the well-established signaling pathways that play a vital role in the modulation of mucosal immunological tolerance relevant to the pathogenesis of IBD is the Janus kinase/signal transducer and activator of transcription (JAK/STAT) system (80). To ensure effective intestinal immunity, the JAK/STAT pathway modulates the proportions of effector to regulatory T cell numbers, intestinal epithelial cells and myeloid cells in the mucosa. In IBD, pro-inflammatory cytokines deliver their signal through cytoplasmic JAKs, which, once phosphorylated, associate with another class of cytoplasmic proteins called STATs. Subsequently, STATs are phosphorylated and translocated into the nucleus, where they enhance the transcription of target genes (including *TGF-β*, *TNF-α*, *IL-2*, *IL-6*, *IL-8*, *MMP9*, *Intercellular Adhesion Molecule 1* (*ICAM-1*/*CD54*), *STAT1*, and *STAT3*) (81).

## 6 Role of antioxidants for IBD-related therapeutic applications and hormonal intervention

Oxidative/nitrosative stress is a significant pathophysiologic factor that plays a role in the initiation and progression of IBD. Overproduction of ROS is stimulated, and consequently, OS is triggered during inflammation because of the large number of cytokines and chemokines secreted by inflammatory cells. Considering this, therapeutic interventions incorporating substances possessing antioxidant and anti-inflammatory properties may be considered. Multiple antioxidant therapeutic strategies are being investigated because of the importance of OS in the pathophysiology of IBD to eliminate ROS, enhance AOE activities, and inhibit abnormal redox signaling (**Figure 2**). Colonic malondialdehyde (MDA) level rises because of OS, LPx, and free radical chain reactions that damage the intestinal mucosal barrier and activate inflammatory mediators. The use of antioxidants in people with uncomplicated GI disorders has been proposed as a potential alternative therapy to the use of anti-inflammatory/immunomodulatory drugs (82). The aim of antioxidant therapies is to mitigate the adverse effects of traditional treatments and to enhance the patient’s quality of life. However, the safety of synthetic antioxidants has been a subject of debate over time, despite their extensive utilization as a viable alternative to natural antioxidants (83–85). Numerous studies have documented a correlation between the prolonged consumption of synthetic antioxidants and certain health problems, such as GI disorders and increased cancer susceptibility (83–85). The utilization of natural antioxidants as a substitute for synthetic products is a noteworthy strategy, given that they are employed within the confines of regulatory thresholds (85).

### 6.1 Phenolic and polyphenolic compounds

Flavonoids, phenolic acids, lignans, and stilbenes are examples of polyphenols, a family of phytochemicals found in many plant diets. A growing body of research has demonstrated that natural polyphenols effectively mitigate the severity of intestinal inflammation and OS in the early stages of IBD (82, 85–89). Polyphenol-rich diets may ameliorate the pathophysiology of disorders where excessive production of ROS plays a role in disease progression (82). The phytochemicals that inhibit Toll-like receptor 4 (TLR4) activation were shown to cause reduced lipopolysaccharide (LPS)-mediated expression of cyclooxygenase-2 (COX-2), NF-κB, and pro-inflammatory cytokines (**Figure 1**; **Supplementary Table 1**). Numerous studies have shown the efficacy of phytochemicals against TLR4-mediated inflammation (**Figure 1**) (137, 138). Flavonoids such as quercetin, catechin, and silymarin have proven therapeutic efficacy in the treatment of IBD (**Figure 3**) (139, 140). Here, they operate as effective antioxidants and cellular modulators of the protein kinase and lipid kinase signaling pathways that drive chronic intestinal inflammation (139, 140). Dogs with idiopathic IBD also have up-regulated activity of the JAK/STAT pathway marked by phosphorylated STAT3 (pSTAT3) overexpression (141). Numerous studies have demonstrated that consuming antioxidants found naturally in plants can neutralize harmful free radicals and protect against certain diseases.

#### 6.1.1 Resveratrol

The polyphenol resveratrol (RSV, trans-3,5,4’-trihydroxystilbene) is found in grapes, soybeans, berries, nuts, and pomegranates, among other natural sources (142). In rodent models of IBD, the antioxidant potential of RSV has been investigated. In one trinitrobenzene sulphonic acid (TNBS)-induced colitis study, pretreatment of rats with RSV reduced histologic inflammation and MDA levels but increased GPx activity compared to markers in the TNBS and vehicle groups (91) (**Supplementary Table 1**).

MPO is responsible for tissue damage in IBD and is inhibited effectively by resveratrol and its derivatives (143). Inhibition of IL-1, IL-6, and TNF-α release from macrophages, iNOS expression and subsequent NO production, prostaglandin production, cyclooxygenase (COX) enzyme activity, apoptosis, and MPO activity are the potential mechanisms by which RSV exerts its anti-inflammatory effect (90, 91) (**Supplementary Table 1**). The action of resveratrol on ROS generation may involve a direct radical scavenger effect or an effect on the activation of NADPH oxidase (90).

It has been shown that the NF-κB pathway is linked to both colitis and colon cancer development as a consequence of chronic intestinal inflammation (144). Additionally, inflammation in the colon inhibits the activity of the silent information regulator 1 (*SIRT-1*) gene and increases the activity of NF-κB. By activating SIRT-1 and down-regulating NF-κB activation, RSV plays a vital role in the regulation of inflammation that mediates colitis and colon cancer (145) (**Supplementary Table 1**).

#### 6.1.2 Curcumin

Curcumin, a polyphenol extracted from *Curcuma longa* rhizomes, has a wide variety of beneficial antioxidant, anti-inflammatory, immunomodulatory, neuroprotective, hepatoprotective, anti-cancer, antiproliferative, and chemopreventive properties (34, 49, 50, 146–148). Curcumin reduces OS and improves intestinal barrier integrity and mitochondrial functions by inducing Parkin-dependent mitophagy via AMPK (adenosine 5’-monophosphate activated protein kinase) activation and TFEB (transcription factor EB) nuclear translocation (149). As a result of its ability to inhibit the expression of transcription factors and pro-inflammatory cytokines such as TNF-α, IL-1, IL-6, IL-8, IL-12, IL-1β, and monocyte chemoattractant protein-1 (MCP-1), curcumin has demonstrated anti-inflammatory properties (**Supplementary Table 1**). Curcumin can also compete with LPS for TLR4 receptor activation, thereby inhibiting the TLR4/myeloid differentiation 88 (MyD88)/NF-κB signaling pathways (150, 151). Curcumin can reduce LPS-induced inflammation in vascular smooth muscle cells via TLR4-MAPK/NF-κB pathways by inhibiting ROS generation (93). This effect is mediated through curcumin’s effects on TLR4 as it specifically prevents the LPS-induced generation of MCP-1, TNF-α, and NO (93). Curcumin reduces TNBS-induced colitis in rats by inhibiting the TLR4/NF-κB signaling pathway and pro-inflammatory IL-27 expression (94).

Oral supplementation with curcumin reduces OS generated by hyperthyroidism in rats, as shown by decreased LPx and protein carbonyl (PC) levels and increased SOD and CAT activities in tissues (34, 39, 49). *In vitro* studies show that curcumin can boost cellular glutathione levels by stimulating the transcription of two Gcl genes (*Gclc* and *Gclm*) encoding glutamate cysteine ligase, which is the rate-limiting enzyme in glutathione synthesis (49, 152). It has been reported that curcumin may reduce OS by modulating Nrf2 and KEAP1 function in the rat heart during altered thyroid states (153). Oral delivery of nanoparticles containing curcumin in dextran sulfate sodium (DSS)-induced colitis in Guinea pigs was associated with a substantial drop in tissue LPx and PC levels, leukocyte infiltration, and TNF-α production (95). Antioxidant balance is also regulated by curcumin in rats with UC by lowering both colonic MPO activity and total NO content as well as increasing colonic GST activity and GSH contents when administered prior to the DSS challenge (154). Curcumin has been shown to decrease OS markers MPO and MDA levels and cell apoptosis in different animal models of colitis (155–158) (**Supplementary Table 1**).

#### 6.1.3 Quercetin

Quercetin (QCT), a member of the flavonols (a subclass of flavonoids), is a polyphenolic molecule found in plants and has demonstrated anti-inflammatory, antioxidant, and antitumoral effects. Several studies have shown that quercetin can suppress the expression of inflammatory mediators and cytokines such as COX-2, NO, NF-kB, prostaglandin E2 (PGE2), iNOS, TNF-α, IL-1β, and IL-6 that are generated through the LPS-TLR4 pathway (159) (**Supplementary Table 1**). LPS-induced inflammation is suppressed by quercetin-rich *Myrsine seguinii* ethanolic extract, which inhibits Src- and Syk-mediated phosphoinositide 3-kinase (PI3K) tyrosine phosphorylation and the TLR4/MyD88/PI3K signaling pathways (160). This extract also suppressed iNOS, a high-output Ca^++^-independent NOS stimulated by cytokines (161), and COX-2 gene expression through reduced NF-κB and activator protein (AP-1) stimulation (160).

Modulation of the stress response genes, including the antioxidant enzyme GPX1, was detected after LPS stimulation of the IBD enteroids and colonoids (57). Organoids have more advantages than conventional models and have been employed in fundamental and clinical research, such as for genetic and infectious diseases, regenerative medicine, and accurate and reliable drug screening (162–168). Murine colitis-derived intestinal organoids stimulated by LPS show reduced mRNA expression of inflammatory mediators such as TNF-α and lipocalin-2 (LCN2) when treated with quercetin (96). The anti-inflammatory action of quercetin was also accompanied by a decrease in the expression of C/EBP-β, a transcription factor that induces the expression of several inflammatory mediators, including TNF-α (169). Human *apoB* promoter analysis shows that a CCAAT enhancer-binding protein (C/EBP)-response element is critical for the action of quercetin. Through its interaction with C/EBPβ, quercetin has the potential to inhibit the recruitment of co-activators. Quercetin also suppresses apolipoprotein B (apoB) expression by inhibiting the transcription of C/EBPβ (169). Several polyphenols, including quercetin, affect the expression of tight junction (TJ) proteins of the intestinal epithelium (88). Using a DSS-colitis murine model, quercetin restored the expression of zonula occludens-1, occludin, junctional adhesion molecule-A, and claudin-3 (88).

There have been several studies on animal models of UC that provide evidence for the use of quercetin to treat IBD (92, 170). Oral quercetin (doses ranging from 25 to 100 mg/kg for 11 days) was associated with decreases in loss of body weight loss, rectal bleeding, and macroscopic and biochemical intestinal damage. It is possible that the antioxidant activity of QCT reduces LPx and OS by regulating nitrites and nitrates, increasing glutathione (GSH), and decreasing MPO activity in the colonic mucosa (92, 170) (**Supplementary Table 1**). Additional research using animal models demonstrates that QCT activity is mediated through the inhibition of TNF-α expression. By regulating the anti-inflammatory effects and bactericidal activity of macrophages through heme oxygenase-1 (HO-1)-dependent pathway, QCT may reduce the severity of experimental colitis (170, 171). Dietary delivery of QCT to restore intestinal homeostasis and intestinal normobiosis is a potentially promising treatment for IBD (171). Quercetin inhibits the apical efflux of N-acetyl 5-aminosalicylic acid (Ac-5-ASA) from Caco-2 cells, which was mediated by multidrug resistance-associated protein 2. This suggests that using QCT as an additional therapy may contribute to reduced dosages of sulfasalazine required for therapeutic action while reducing adverse drug effects (172). In another murine colitis model study (173), quercetin-loaded microcapsule-treated mice showed significantly more 2,2’-Azino-bis (3-ethylbenzothiazole-6-sulfonic acid) (ABTS) radical cation scavenging and ferric reducing activity as compared to colitis control mice. Results indicated that the QCT-treated mice had significant reduction in neutrophil influx (MPO activity), edema, and colonic macroscopic and histologic inflammation. Finally, this treatment reduced the levels of the pro-inflammatory cytokines IL-1β and IL-33 while maintaining anti-inflammatory cytokine IL-10 levels, and maintained the levels of endogenous antioxidants in the colons of colitic mice (173)(**Supplementary Table 1**).

#### 6.1.4 Green tea flavonoids

Currently, there is no established tolerable upper limit for flavonoids in the Dietary Reference Intake framework (174). However, the consumption of flavonoids in quantities naturally present in foods does not pose any toxicity concerns (175). A high consumption of flavonoids may increase the likelihood of iron deficiency in populations with suboptimal iron levels (e.g., the elderly). Nonetheless, in Western societies where sufficient intake of heme iron and ascorbic acid occurs, the likelihood of developing anemia is minimal (175). The bioavailability of flavonoids is limited, as only a small fraction (i.e., less than 10% of the ingested quantity) can attain peak concentrations in the bloodstream within a few hours The bioavailability of flavonoids may be subject to various factors, such as ingestion of dietary fiber, macro and micronutrients, the duration of GI transit times, and composition of the gut microbiota (176, 177).

Flavonoids in green tea have been shown to regulate the expression of pro-inflammatory genes that target TLRs and inhibit downstream MyD88- and Toll/IL-1R domain-containing adaptor-inducing IFN-β (TRIF)-dependent signaling pathways (89). Epigallocatechin-3-gallate (EGCG), a flavonoid present in green tea, reduces TNF-α and also gene expression and the effects of nitric oxide synthase (NOS) and COX in murine RAW 264.7 macrophages (97). Treatment with EGCG-docosapentaenoic acid (DPA) esters has been shown to reduce the production of the pro-inflammatory mediators like nitric oxide (NO) and PGE2 via down-regulation of iNOS and COX-2 gene expression. Other EGCG ester derivatives (stearic acid, eicosapentaenoic acid, and docosahexaenoic acids) also have anti-inflammatory activity in murine RAW 264.7 macrophages (97). The mechanism of action is believed to be the inhibition of downstream TLR signaling affecting the MyD88- and/or TRIF-dependent pathways with activation of NF-κB. A study also demonstrated that EGCG blocks both the MyD88-dependent and TRIF-dependent signaling pathways of TLRs in RAW264.7 cells (89). In TRIF-dependent signaling pathways of TLR3 and TLR4, the molecular target of EGCG is TANK-binding kinase1 (TBK1), resulting in the decrease of interferon regulatory factor 3 (IRF3) activation, as TBK1 is the downstream kinase of TRIF and phosphorylates IRF3 resulting in its activation (89).

The combination of EGCG and piperine (piperine for enhancing the bioavailability of EGCG) significantly decreased weight loss, improved the clinical course, and increased overall survival in the DSS-murine model of colitis (87). Reduced severity of the colitis was linked to improved histology scores and reduced colonic MDA and MPO activity (**Supplementary Table 1**). The combination of EGCG and piperine improved SOD and GPx expression and suppressed the generation of proinflammatory cytokines *in vitro*. It appears that the powerful antioxidative potential of EGCG is responsible for its anti-inflammatory effects in the DSS-murine model of colitis. Lipid peroxidation occurs during IBD inflammation when ROS are not neutralized, altering the permeability and selectivity of the cell membrane and the activity of transmembrane transporters, receptors, and enzymes (87). DSS-colitis mice that had been administered EGCG and piperine had increased levels of AOEs (SOD and GPx), indicating that the antioxidant capacity had improved (87) (**Supplementary Table 1**).

#### 6.1.5 Caffeic acid phenethyl ester

The anti-inflammatory, anti-cancer, and antioxidant effects of caffeic acid phenethyl ester (CAPE) have been studied extensively (98, 99, 178–181). Caffeic acid phenethyl ester inhibits LPS-induced IL-12 production and NF-κB activation in monocyte-derived dendritic cells (178). Caffeic acid phenethyl ester prevents the activation of TLR4 by interfering with the interaction between the TLR4/MD2 complex (180). In LPS-induced breast cancer cells, CAPE can down-regulate the expression of TLR4, NF-kB p65, TRIF, MyD88, and IRAK4 while stimulating cell apoptosis and autophagy (181). In gingival fibroblasts, CAPE suppresses LPS-induced production of IL-6, IL-8, iNOS, COX-2, TLR4/MyD88 mediated NF-κB, and phosphorylation of PI3K and protein kinase B (PKB, or Akt) (179).

FA-97 (caffeic acid phenethyl ester 4-O-glucoside) is a new synthetic CAPE derivative shown to attenuate body weight loss, colon length shortening, increased colonic inflammatory cell infiltration, and pro-inflammatory cytokine production (99). While FA-97 increased overall antioxidant capacity in DSS-treated mice and LPS-treated BMDMs and RAW 264.7 cells, it also decreased ROS and MDA production. The mechanism of action of FA-97 is believed to be the activation of the Nrf2/HO-1 pathway in both *in vivo* and *in vitro* models. FA-97 activates Nrf2 and promotes its nuclear translocation to increase expression of its downstream target proteins HO-1 and NAD(P)H:quinone oxidoreductase (NQO-1), which reduces ROS. It also inhibits the NF-κB and AP-1 signaling to suppress the expression of pro-inflammatory cytokines IL-1, IL-6, TNF-α, and IL-12 (**Supplementary Table 1**). In addition to ameliorating DSS-induced colitis, FA-97 promotes normal epithelial barrier function (98, 99).

#### 6.1.6 Luteolin

The flavonoid compound luteolin, found in various plant extracts, has been shown to have anti-inflammatory, antioxidant, anti-metastasis, and apoptosis-inducing properties. Luteolin has been reported to reduce LPS-stimulated expression of NF-κB, TNF-α, and ICAM-1 and TBK1-kinase activity via the MyD88-independent signaling pathway (182). Luteolin inhibited the expression of target genes IL-6, IL-12, IL-27, TNF-α, IP-10, IFNβ, CXCL9 (C-X-C motif chemokine ligand 9) in macrophages by inhibiting IRF3 and NF-κB activation (**Supplementary Table 1**). Luteolin was able to suppress the ligand-independent activation of IRF3 or NF-κB triggered by TLR4, TRIF, or TBK1. Luteolin also reduces the amount of TBK1-dependent gene expression by inhibiting TBK1-kinase activity, and IRF3 dimerization and phosphorylation. Moreover, luteolin structural analogs, such as quercetin, chrysin, and eriodictyol, also inhibit TBK1-kinase activity and TBK1-target gene expression. These findings indicate that TBK1 is a unique target of anti-inflammatory flavonoids resulting in the inhibition of the TRIF-dependent signaling pathway (182).

LPS-induced inflammatory responses are controlled at the transcriptional level through the MAPK and NF-κB pathways (100, 183, 184). Toll-like receptors trigger NF-κB and MAPK cascades responses to LPS stimulation, causing the production of ROS, increased MPO expression, and expression of pro-inflammatory molecules and chemokines. In mice with LPS-induced acute lung injury (ALI), luteolin reduced the activation of ERK, p38MAPK, and JNK in lung tissue. The protective action of luteolin is due to its ability to block MAPK pathways, which prevents the activation of NF-κB and the degradation of IκB. Upon administration of LPS, luteolin pretreatment prevents these inflammatory processes from developing (100). In addition, the results in mice indicate that the activities of SOD and CAT increased following pretreatment with luteolin versus LPS treatment alone. MPO levels are reduced in LPS-induced acute lung injury (ALI) when pretreated with luteolin. This mechanism of action is believed to be due to luteolin suppression of LPS-induced ALI-related MDA generation in the lungs (100) (**Supplementary Table 1**).

#### 6.1.7 Xanthohumol

Xanthohumol (XN), a compound derived from hop plants, is a prenylated chalcone with, antioxidant, anti-cancer, and anti-inflammatory activities via inhibition of TLR4/MD-2 complex (185). Downstream, XN suppresses macrophage iNOS expression and NO, and interferon-gamma (IFN-γ) production (186, 187). Pretreatment with XN in DSS-treated mice decreased the severity of diarrhea, hematochezia, rectal bleeding, and reduced colon length. Moreover, XN protected against epithelial cell injury, cellular infiltration, pro-inflammatory cytokines, and crypt changes following the DSS challenge (101). Pre-treatment with XN prior to DSS exposure resulted in decreased MDA levels and COX-2 expression suggesting a protective effect against experimental colitis (101) (**Supplementary Table 1**). Moreover, nuclear factor of kappa light polypeptide gene enhancer in B-cells inhibitor, alpha (IκBα) phosphorylation, nuclear translocation of p65, p50, and p105 NF-κB subunits, and NF-κB DNA-binding transcriptional activity were suppressed by XN treatment of DSS-treated mice and H_2_O_2_- or LPS-treated IEC-6 cells (101).

XN protects mice from DSS-induced colitis and H_2_O_2_- or LPS-treated IEC-6 injury, possibly by the interaction between the α, β-unsaturated carbonyl moiety of XN and Cys99 in IKKβ, and thus by its ability to inhibit the IKKβ/NF-κB signaling pathway. Additionally, XN inhibited the activation of the canonical NF-κB pathway, as evidenced by the downregulation of NF-κB target genes, like *A1a*, *A20*, *Bcl-xL*, and *c-myc* (101) (**Supplementary Table 1**). These collective results indicate that XN may be a promising therapeutic agent for the prevention or treatment of colitis (188).

#### 6.1.8 Genistein

A soy isoflavone, genistein, is a powerful antioxidant and anti-inflammatory agent (102–105, 189). Following treatment with genistein, reduced levels of IL-6, TNF-α, and NF-κB activation have been observed in *in vitro* and *in vivo* studies (104, 189). Genistein also inhibited BV2 microglia LPS-induced NO production, prostaglandin E2 release and expression of inflammatory cytokines (IL-1β, TNF-α), TLR4, and MyD88 expression (102). Expression of interferon beta (IFN-β), IL-1α, IL-1β, IL-6, IL-10, TNF-α, colony-stimulating factor 2 (CSF-2), colony-stimulating factor 3 (CSF-3), chemokines CCL2 (chemokine ligand 2), and CXCL10 (C-X-C motif chemokine ligand 10), transcription factor NF-κB, IκBα, and COX-2 are all decreased in genistein-pretreated LPS-induced RAW264.7 macrophages (103) (**Supplementary Table 1**). In humans, six months of daily oral administration of genistein has been shown to decrease TNF-α levels decreased in obese postmenopausal women (106). Dietary supplementation with genistein lowered the expression of vascular adhesion molecule-1 (VCAM-1), a major cell adhesion molecule involved in inflammation (190), and F4/80 positive macrophages in the aorta of TNF-α-treated C57BL/6 mice (104). Human umbilical vein endothelial cells (HUVECs) in culture showed significant increases in the activities of GR, GPx, GST, NQO-1, SOD, and CAT when treated with 50 µM genistein (104) (**Supplementary Table 1**). Due to its ability to stimulate IL-1β secretion, the NLRP3 inflammasome has been linked to IBD in experimental models as well as human studies (191). In the DSS murine colitis model, treatment with genistein significantly slowed weight loss and reduced colon shortening, infiltration of inflammatory cells as well as the production of pro-inflammatory mediators in both serum and colon (105). Genistein’s protective properties may stem from the ubiquitination of NLRP3 caused by the interaction of cAMP with the protein. It has been shown that genistein can inhibit the NLRP3 inflammasome via TGR5-cAMP signaling in human macrophages (105).

#### 6.1.9 Alpinetin

Alpinetin, a naturally occurring dihydroflavone (192), ameliorates clinical severity (disease activity index; DAI), colonic shortening, histological scores, and MPO activity in DSS-treated mice (108). The expression of occludin and zonula occludens-1 and SOD activity were increased, while the MDA amount was decreased by alpinetin in DSS-treated mice. Also, Nrf2/HO-1 signaling pathways were activated by alpinetin in DSS-treated mice (108). Alpinetin exhibits its anti-inflammatory activities by suppressing TLR4/IκBα/NF-κB signaling (107). In that study, alpinetin decreased the expression levels of IL-1β, IL-6 and TNF-α (**Supplementary Table 1**) in LPS-treated RAW 264.7 macrophages *in vitro* and in the *in vivo* LPS-induced acute lung injury murine model (107) by disrupting ERK/p38MAPK signaling (193).

#### 6.1.10 Proanthocyanidins

Grape seed proanthocyanidin extract (GSPE) is composed of approximately 90% proanthocyanidins, including oligomers (74.8%), dimers (6.6%), trimers (5.0%) and tetramers (2.9%) (194). Inhibiting the expression of NF-κB-targeted genes is a primary mechanism by which GSPs exert their antioxidant and anticarcinogenic actions (109, 195, 196). It is proposed that GSPs inhibit NF-κB activation by inhibiting the activation of IκK (inhibitor of κB kinase) to prevent phosphorylation-induced degradation of IκBα (196). The effect of GSPs on enhancing antioxidant enzymatic defense was confirmed by their ability to boost colonic SOD activity (109, 195).

Nutritional and pharmacological dosages of proanthocyanidins protect against endotoxin (LPS)-induced intestinal inflammation, OS, and intestinal permeability in colitic rats (197). Notably, area-specific LPS-induced inflammation in the intestine has been identified, and a unique gene signature may be useful to determine the affected intestinal region (57). Treatment with GSPE reduces MPO and COX-2 activity, modifies the gene expression of ileal inflammatory and permeability proteins, and reduces ROS, MDA, and NO levels, and iNOS activities (**Supplementary Table 1**) in the large intestine (109, 197). Also, GSPE treatment enhances SOD, GPx activity, and glutathione levels in colon tissues and serum of TNBS-induced colitis in rats (109) (**Supplementary Table 1**).

#### 6.1.11 Anthocyanins

The antioxidant properties of anthocyanins, as well as their ability to influence gut microbiota and to down-regulate the immune response, have significant implications for reducing intestinal inflammation (198, 199). In human monocytic THP-1 cells, treatment with bilberry extract (BE), rich in anthocyanins, inhibited IFN-γ activation of STAT1 and STAT3 and lowered mRNA expression and/or secretion of MCP-1, TNF-α, IL-6, and ICAM-1 (110). The effects of genetically engineered tomato extracts (enriched in anthocyanins) (200) showed that the extract reduced the activation of epithelial cells SAPK/JNK (stress-activated protein kinase/c-Jun NH (2)-terminal kinase) and p38MAPK signaling pathways. Furthermore, pro-inflammatory cytokines and chemokines, including TNF-α and IL-10, were inhibited by anthocyanin-rich tomato extracts (200). It has been shown that anthocyanin-rich fractions from red raspberries (112) and purple carrots (111) decrease the production of iNOS, COX-2, IL-1β, and IL-6 in LPS/IFN-γ activated macrophages *in vitro*. In murine colitis, anthocyanins extracted from the Chinese plant *Dioscorea alata* L (201). decreased DAI (body weight loss, fecal occult blood, and fecal consistency), increased the gene expression of tight junction proteins and reduced OS markers (MPO and iNOS) along with IFN-γ and TNF-α levels. Red raspberry berry (RB) powder (202) can attenuate the effects of DSS treatment by preventing weight loss, neutrophil infiltration, colon shortening by inhibiting IL-1, IL-6, IL-17, and TNF- α and COX-2 levels in inflamed tissues (**Supplementary Table 1**). Supplementation with RB restored CAT levels to normal, decreased xanthine oxidase (XO) levels and expression of MCP-1, and thus reduced neutrophil infiltration and ROS formation (202). Similarly, when an extract of blueberry anthocyanins (BBA) (203) was used, disease activity, MPO and MDA levels were reduced in murine colitis. In addition to decreasing serum prostaglandin E2 levels, BBA reduces OS marked by increased SOD and CAT levels. Moreover, mRNA expression of NF-κB, IFN-γ, COX-2, IL-1β, and iNOS was reduced, indicating that blueberry’s protective effect is at least partially mediated by the inhibition of inflammatory mediators (203). Similarly, it has been shown that colitis symptoms (colon shortening, DAI, loss of appetite, and weight gain), decreased SOD, and increased MPO can be mitigated and that GPx and GR activities (**Supplementary Table 1**) can be increased by administering grape pomace extract (GPE) (86). Anti-inflammatory effects of GPE were observed in DSS-treated mice as evidenced by reduced IL-1β, IL-1α, IL-6, and IFN-γ and also TNF-α activities. Gene expression for the p65 subunit of NF-κB, TNF-α, and COX-2 was reduced, which down-regulates the production of pro-inflammatory cytokines by GPE (86). Similarly, cocoa (anthocyanidins/anthocyanins) supplementation (204) decreased MDA, increased SOD, CAT, GPx, and GRx, and reduced iNOS and COX-2 gene expression in murine colitis. Moreover, cocoa is able to stimulate Nrf-2 transcription factor-activated expression of NQO1 and UDP-GT to reduce OS. Cocoa supplementation can also lower CD68^+^ neutrophils, MPO, TNF-α, IL-1β and IL-17 and suppress STAT-3 activation in colitic tissues (204) (**Supplementary Table 1**).

One randomized, placebo-controlled trial involving healthy humans found that eating anthocyanins-rich, purple-fleshed potatoes for six weeks reduced the levels of inflammatory markers like IL-6 and C-reactive protein (CRP) and the oxidative DNA-damage marker 8-hydroxydeoxyguanosine (8-HdG) (205). In another study, patients with mild to moderate UC were given an anthocyanin-rich bilberry preparation for six weeks and showed improvements in endoscopic Mayo score, histologic Riley index, and fecal calprotectin levels for intestinal inflammation (206).

#### 6.1.12 Silymarin

Silymarin (SM), an extract from milk thistle (*Silybum marianum*), has been shown to have anti-inflammatory and antioxidant properties. Silymarin decreases inflammation by inhibiting NF-κB pathways and by optimizing the redox balance in the cell through activating AOEs and non-enzymatic antioxidants via Nrf2 activation (207). Studies show that SM increases the total antioxidant capacity of colonic tissue to reduce LPx levels, neutrophilic infiltration and pro-inflammatory cytokine production in TNBS-induced colitic rats. Also, treatment with SM in TNBS-colitic rodents dramatically decreased colonic NF-κB activity, levels of IL-1β, TNF-α, TBARS and MPO activity (113) (**Supplementary Table 1**). In one randomized, double-blind, placebo-controlled clinical trial, 35/38 UC patients administered SM for 6 months achieved remission. Moreover, remission was accompanied by improved patient biochemical parameters such as decreased erythrocyte sedimentation rates, increased hemoglobin levels, and decreased DAI scores (208). However, the bioavailability of oral SM appears to vary widely between species and is impacted by preparation methods (209–211). Thus, care should be taken when designing experimental trials and extrapolating results between species and preparations.

#### 6.1.13 Thymol

Essential oils from Thymus, Origanum, and Lippia species are rich in thymol (2-isopropyl-5-methylphenol), a monoterpene phenol derivative of cymene (212). Thymol has demonstrated a wide range of biological and pharmacological activities, such as anti-inflammatory, antioxidant, anti-tumor, and antimicrobial effects (213). Thymol exhibits gastroprotective effects on both acute and chronic ulcer models by modulating the enhancement of mucus production, prostaglandin synthesis, and activation of ATP-sensitive K(+) channels (212). Thymol mitigates the reduction of trans-epithelial electrical resistance (TEER) in a porcine IPEC-J2 monolayer cell model stimulated with LPS by increasing ZO-1 and actin expression (118). In the DSS-induced murine colitis model, thymol mitigates intestinal damage induced by DSS by up-regulating the expression of tight junction protein claudin-3 (119). Similarly, thymol enhances the tight junction integrity and induces up-regulation of cyclooxygenase-1 (COX1) activity in Caco-2 cells (115).

Along with improving barrier function, thymol can decrease the production of ROS and the expression of pro-inflammatory cytokines following stimulation with LPS (118). Thymol exhibits the ability to attenuate increased MPO and MDA levels induced by LPS, as well as the expression of NF-κB, in a murine model of acute lung injury (214) and in colonic homogenates in murine colitis (215). Thymol exhibits inhibitory effects on the expression of TLR4 and the activation of NF-κB signaling in mice treated with acetic acid to induce colitis (117, 120). Thymol has been found to exhibit inhibitory effects on p38 phosphorylation, and it disrupts the activation of the MAPK signaling pathway, thereby contributing to the maintenance of immune homeostasis (216). Moreover, thymol exhibits inhibitory effects on the activation of p-p38, p-JNK, and p-ERK induced by LPS in RAW264.7 cells (116). Consequently, thymol effectively suppresses the production of various inflammatory cytokines such as NO, IL-6, TNF-α and COX-2 (116), thus exhibiting the ability to attenuate the inflammatory response through the inhibition of MAPK signaling pathway. Similarly, thymol had the potential to mitigate inflammatory responses by regulating the expression of JNK, AP-1, STAT-3, and nuclear factors of activated T-cells (NFATs) in LPS-stimulated J774.1 mouse macrophages (117).

### 6.2 Alkaloids

#### 6.2.1 Berberine

Berberine, an isoquinoline alkaloid from *Berberis aristata*, has been used for decades to treat intestinal parasites and enteropathogenic diarrhea due to its bactericidal activity, its ability to inhibit protozoan growth, and also to inhibit enterotoxin-induced intestinal electrolyte secretion (217–219). Several *in vivo* studies confirm its anti-inflammatory role in decreasing the expression of IL-1β, TNF-α, iNOS, ICAM-1, IL-6, and NF-κB activation (220) (**Supplementary Table 1**). Berberine reduces damage, inflammation scores, MPO activity, and colon shortening caused by oral DSS ingestion. Moreover, berberine has been shown to decrease levels of the pro-inflammatory cytokines IFN-γ, TNF-α, KC (keratinocyte chemoattractant or CXCL1), and IL-17 and to maintain colon epithelial barrier function in DSS-treated mice. Additionally, berberine enhanced apoptosis of colonic macrophages and decreased proinflammatory cytokine production in colonic macrophages and epithelial cells of DSS-treated mice. Berberine suppresses Src activation and TLR4-mediated cell motility in LPS-stimulated macrophages (121). Berberine also inhibits the activation of MAPK and NF-κB signaling pathways that stimulate proinflammatory cytokine production in both colonic macrophages and epithelial cells from DSS-treated mice (122). Multiple cellular kinases and signaling pathways, including AMPK, MAPKs, and Nrf2 and NF-κB pathways, are involved in berberine’s antioxidant and anti-inflammatory activities (221). By suppressing the expression of NADPH oxidase, a key enzyme in the generation of ROS in cells, berberine can mitigate OS (222). Superoxide anion production in LPS-stimulated macrophages was suppressed, while SOD activity normally was restored following berberine treatment. Since it can suppress gp91phox (a plasma membrane subunit of NADPH oxidase) expression and boost SOD activity, berberine is able to restore cellular redox activity (222) (**Supplementary Table 1**). In this instance, it is possible that berberine enhances SOD expression through the silent mating type information regulation 2 homolog 1 (SIRT1)/Forkhead Box Class O (FOXO) pathway (221). In other experiments, berberine has been shown to directly bind to PLA2G4A and inhibit the MAPK/JNK signaling pathway to suppress PLA2G4A activity in murine colitis (223). A double-blind placebo-controlled phase I trial (123) demonstrated that berberine reduced colonic tissue inflammation (Geboes score) (224) in mesalamine-treated UC patients. However, it had no effect on inflammatory biomarkers in other tissues or blood (123).

### 6.3 Storage polysaccharides

#### 6.3.1 Tamarind xyloglucan

Tamarind xyloglucan (TXG), a nanofiber extracted from tamarind, is a novel antioxidant that prevents DSS-induced colitis in mice (124, 125). TXG protects the colon by reducing the total inflammatory index, infiltration of inflammatory cells, submucosal edema, goblet cell loss, epithelial erosion, granulation tissue, epithelial hyperplasia, and crypt irregularity, abscesses and loss (124, 125). While TXG reduces inflammation by reducing TNF-α and increasing anti-inflammatory IL-10, it reduces OS by lowering levels of MDA, superoxide anion production, and the expression of iNOS, NOX, COX-2, and p47 (125) (**Supplementary Table 1**).

### 6.4 Other phytochemicals

#### 6.4.1 Sulforaphane

Sulforaphane, found in cruciferous vegetables, contains a wide range of antibacterial, antioxidant, anti-inflammatory, and immunomodulatory properties (225, 226). Treatment of rats with colitis using sulforaphane reduces NO and MDA levels, accompanied by increased GPx and reduced glutathione levels, demonstrating its therapeutic antioxidant properties (8). Treatment with sulforaphane increases the levels of Nrf2 and HO-1 (8) (**Supplementary Table 1**). It has been observed that HO-1, an antioxidant defense protein downstream of Nrf-2, prevents oxidative damage to colonic tissue (99). Sulforaphane possesses anti-inflammatory characteristics by decreasing TNF-α and IL-6 levels, inhibiting COX-2 expression, TLR4 oligomerization, TLR4/MyD88 pathway and blocking the degradation of IL-1R-associated kinase-1, NF-κB, and IFN regulatory factor 3 activation (227, 228).

### 6.5 Food/spices

#### 6.5.1 Flaxseed oil (α-linolenic acid)

Flaxseed oil is an herbal product with a high α-linolenic acid content, and it has been shown to reduce colonic damage in DSS-induced colitis by modulating inflammatory factors, oxidative state, and the cecal microbiota imbalances. DSS-colitic rat colon showed low SOD activity and GSH levels, increased MPO activity and MDA levels, and flaxseed oil dose-dependently alleviated this condition. Compared to the DSS-treated group, flaxseed oil treatment for six weeks raised SOD activity and GSH levels while decreasing MDA levels and MPO activity (127) (**Supplementary Table 1**). A metabolite of linoleic acid, 10-hydroxy-cis-12-octadecenoic acid (HYA), inhibits TNF-α expression and DSS-induced alterations in the expression of TJs such as occludin, zonula occludens-1, and myosin light chain kinase. The metabolite HYA partially restores the integrity of the intestinal epithelial barrier via the GPR40-MEK-ERK pathway, as reported in human Caco-2 cells and the murine DSS-colitis model (126).

#### 6.5.2 Ginger

Ginger rhizomes, a rich source of many active compounds, including gingerols, shogaols, gingediols, zingerone, dehydrozingerone, gingerinone, and diarylheptanoids, have a wide range of anti-inflammatory, analgesic, antioxidant, and anti-cancer effects (129, 151, 229). S-[6]-gingerol inhibits the expression of the inflammatory mediators IL-6, IL-8, and serum amyloid A1 (SAA1) in cytokine-stimulated human HuH7 hepatocyte cells by inhibiting the NF-κB/COX-2 pathway to reduce OS (129). Concentration-dependent inhibition of LPS-induced IL-1β, IL-6, TNF-α and PGE2 levels was demonstrated with 6-shogaol, the most bioactive component of ginger (230). 6-Shogaol also reduces the phosphorylation and nuclear translocation of NF-κB p65, thus preventing LPS-induced NF-κB activation. In LPS-induced murine RAW 264.7 macrophages, 6-shogaol was reported to inhibit protein and mRNA expression of iNOS and COX-2 (128). By inhibiting the phosphorylation of inhibitor κB (IκB)α and p65 and the subsequent degradation of IκBα, 6-shogaol decreased the LPS-induced activity of NF-κB. PI3K/Akt and extracellular signal-regulated kinase 1/2 activation by LPS is also blocked by 6-shogaol, although the p38MAPK activation is not (128). Since TLR4 dimerization mediated by LPS is necessary for the activation of downstream signaling pathways, including NF-κB, 6-shogaol blocks this process and prevents NF-κB activation (231). Also, 6-shogaol can block TLR-mediated signaling pathways directly at the receptor (231). In LPS-induced macrophages, 6-shogaol suppresses the MyD88-dependent signaling pathway by inhibiting IκB kinase activity and TRIF-dependent signaling pathways that target TBK1 (232). Compounds extracted from ginger were tested for their ability to stimulate phagocytosis and suppress nitric oxide generation in the RAW 264.7 cell line induced by LPS (229). Significant reductions in both LPS-induced nitric oxide production and inducible nitric oxide synthase expression were observed when 6-shogaol, 1-dehydro-10-gingerdione, and 10-gingerdione were applied (229). Macrophages are essential components of the immune system as they are responsible for the elimination of necrotic cells. This function is critical as it prevents the release of harmful contents from these cells, thereby minimizing a proinflammatory response (233). RAW 264.7 cells treated with 1-dehydro-10-gingerdione (1D10G) showed increased phagocytic activity similar to stimulation by LPS. Also, RAW 264.7 cells treated with 6-gingerol, 8-gingerol, 10-gingerol, 6-paradol, 10-gingerdione, 1,7-bis-(4’ hydroxyl-3’ methoxyphenyl)-5-methoxyhepthan-3-one, and methoxy-10-gingerol exhibited increases in phagocytic activity (229). 1D10G directly decreased the catalytic activity of cell-free IκB kinase β (IKKβ) in RAW 264.7 macrophages activated with the TLR4 agonist LPS. In LPS-activated macrophages, 1D10G inhibited TLR4-mediated expression of TNF-α, NF-κB, IL-1β, IRF3, IFN-β and IP-10 (234). Furthermore, in macrophages triggered by the TLR agonists LPS or TNF-α, D10G irreversibly blocks cytoplasmic IKKβ-catalysed IκBα phosphorylation and IKKβ vector elicited NF-κB transcriptional activity to minimize inflammatory signals (235). Substitution of Cys (179) with Ala in the activation loop of IKKβ abrogates these effects suggesting a direct interaction site of D10G. Lastly, D10G reduced NF-κB activation in LPS-stimulated macrophages and decreased the expression of iNOS, COX-2, and IL-6 (235).

In mice with DSS-induced colitis, ginger alleviates the pathological lesions and reduces the expression of IL-6 and iNOS (131). This study also showed that ginger has an anti-inflammatory effect like that of the anti-inflammatory medication sulfasalazine (SASP), reducing DAI and preventing further weight loss (131). Ginger reduces IBD activity by targeting IL-17, IFN-γ, and TNF-α, while increasing the anti-inflammatory cytokines IL-10, IL-22, and TGF-β (236) (**Supplementary Table 1**).

Ginger extract exhibited the antioxidant effects in IL-1β-mediated OS in human C28I2 chondrocyte cells by stimulating the expression of several AOEs, decreasing IL-1β-induced ROS production, LPx, and by reducing apoptosis (Bax/Bcl-2 ratio, and caspase-3 activity) (130) (**Supplementary Table 1**). Patients with active mild to moderate UC who ingested 2000 mg/day of dried ginger powder in a randomized, placebo-controlled clinical trial had lowered MDA without affecting serum total antioxidant capacity (132).

### 6.6 Hormones and their anti-inflammatory and antioxidant roles in IBD

There is growing recognition that the endocrine system plays an important role in the pathogenesis of IBD through various mechanisms (237). Hormone actions influence seemingly every phase of inflammatory and immunological responses, and the intestinal tract is the largest endocrine gland of the body, secreting a vast amount of peptides with paracrine or endocrine function. In sites of inflammation, several hormone receptors have been found to be present in the reactive structures, which are known to have both pro- and anti-inflammatory effects. Signals are generated upon the binding of hormone molecules to specific hormone receptors; these receptors are found on the surface of endothelial and inflammatory cells and play a role in both pro- (such as insulin receptors) (238, 239) and anti-inflammatory (glucocorticoid receptors) (240–242) responses, respectively. The activity of the adrenal cortex is responsible for mediating the indirect anti-inflammatory effects that are caused by glucagon and thyroid hormones (243). Therefore, inflammation is not only a local response but also a hormone-controlled process that occurs locally (paracrine regulation) and throughout the body (endocrine modulation).

One study involving 1,203 females (64% diagnosed with CD,34% diagnosed with UC) reported increased symptoms during their menstrual cycle. Symptoms were comparable among CD and UC cohorts except for pregnant women, where symptoms worsened (244). Women with UC have increased symptoms as compared to women with CD. Understanding the significance of hormones in the context of IBD is crucial for identifying potential approaches to managing hormonal-associated symptoms in women with IBD. The primary endocrine manifestations of IBD include growth failure, metabolic bone disease, alterations in lipid and carbohydrate metabolism, pubertal delay, and hypogonadism (237). These manifestations are interrelated, and their complex development is influenced by intestinal inflammation and the individual’s nutritional status.

#### 6.6.1 Sex hormones

Estrogens have been shown to increase AOEs by upregulating their expression (34, 147, 245). The antioxidant impact of estrogen is thought to be the principal method by which this hormone protects various tissues from oxidative damage (246–248). It has been postulated that sex hormones such as estrogen, progesterone, and androgen contribute to the pathophysiology of sexual dimorphism in human IBD (249). These control the behavior of many different types of immune cells, including lymphocytes, macrophages, granulocytes, and mast cells. Multiple clinical features of the disease, including intestinal barrier disintegration and mucosal immune activation, may be modulated by sex hormones, as shown in both clinical and experimental models (249). There is growing interest in the potential regulation of the intestinal microbiota by sex hormones. Estrogens have an effect on the microbicidal activity that is carried out by MPO in polymorphonuclear leukocytes (250). In CD, the G protein-coupled estrogen receptor (GPER) appears to be a powerful therapeutic target in maintaining remission because it promotes anti-inflammatory effects. Reducing mortality, improving macroscopic and microscopic scores, and lowering CRP levels were all achieved with GPER activation in a TNBS-induced CD murine model (251). Immunohistochemistry verified that estrogen signaling inhibits intestinal inflammation. Genes involved in signal transduction and immunological response, as well as the expression of certain miRNAs (miR-145, miR-148-5p, and miR-592), were shown to be altered in tandem with GPER activation, as was the extracellular-signal-regulated kinase (ERK) signaling pathway (251).

Increased expression of tight junction proteins was a mechanism by which progesterone and estrogen facilitated wound healing and epithelial barrier function in intestinal epithelial cells, as reported using 2D cell lines and IBD patient-derived inflammatory organoid models (252). These sex hormones also inhibited the generation of pro-inflammatory cytokines in intestinal epithelial models and greatly decreased endoplasmic reticulum (ER)-stress. Pregnancy hormones like estrogen and progesterone have been shown to have beneficial effects on disease activity by positively modulating the intestinal epithelial lining (252).

#### 6.6.2 Glucagon

Glucagon decreased iNOS expression and plasma levels of nitrite/nitrate in LPS-treated rats (253). Glucagon is responsible for inducing the antioxidant response by increasing GSH levels and reducing both protein carbonyl and 3-nitrotyrosine (254) or by upregulating AOEs as indicated by high levels of CAT expression in the α cells of diabetic vs. non-diabetic murine models (255).

#### 6.6.3 Enterohormones

Glucagon-like peptide-1 (GLP-1) and GLP-2 are important enterohormones that mediate local and systemic effects (gut-brain-periphery axis), contribute to glucose homeostasis, and modulate GI functions such as the intestinal absorption of lipids and antioxidant defense (256). The effects of these molecules critically depend upon nitric oxide synthase (NOS) in the enteric nervous system (ENS) and the intestinal microbiome (257).

Activation of the GLP-1 receptor has anti-inflammatory, antioxidant, and anti-apoptotic effects, which include a reduction of the pro-inflammatory actions of advanced glycation end products (258) and of the accumulation of intracellular ROS, the release of NO, and GPx and SOD production (259). Moreover, it has been proposed that GLP-1 plays a critical role in the production of NO (260), and intestinal GLP-1 production can be increased by the ingestion of grape-seed procyanidin extract (GSPE), resveratrol, and curcumin (261–264). GSPE also increases intestinal peptide YY (PYY) and varied cholecystokinin (CCK) secretion, which modulates food intake (261, 265, 266).

The enterotrophic peptide hormone glucagon-like peptide 2 (GLP-2) was shown to abrogate OS and improve intestinal antioxidant capacity by reducing LPS-induced increases in intestinal IL-1β and oxidized glutathione levels (267) and increasing intestinal SOD activity and reduced-glutathione levels (268, 269) (**Supplementary Table 1**). Additionally, GLP-2 has a protective effect on the function of the intestinal barrier (270), intestinal IgA production (270), NO-regulated intestinal perfusion (271), and stabilizes the expression and function of intestinal xenobiotic transporters (267). Because intestinal barrier function is critically impaired in IBD and CIE, complex neutraceuticals that provide antioxidants and can enhance intestinal GLP-2 secretion, and thus support enterocyte tight junctions and intestinal barrier integrity (e.g., berberine, soy flavonoids, pre-/probiotics, soluble fiber, glutamine), may be useful to restore and maintain intestinal health (272).

Given the effects of GLP-1 and GLP-2 in maintaining intestinal homeostasis, including cross-talk with the immune and central nervous systems (273, 274), they appear to present potentially beneficial treatment targets in IBD and CIE.

Reduced levels of serum motilin and gastrin, together with lower pro-inflammatory cytokines and reduced tissue NF-κB and COX-2 levels, and increased serum somatostatin, vasoactive intestinal peptide, and tissue SOD, NO, and MDA levels were associated with genistein- and daidzein-rich fermented soy (shuidouchi) in an experimental animal model (275). Similar antioxidative, anti-inflammatory, and enterohormone signature-modulating effects have been demonstrated in experimental mice receiving unsaturated fatty acid-rich silkworm pupa oil (276).

#### 6.6.4 Glucocorticoids

The presence of glucocorticoid receptors in endothelial and inflammatory cells (240–243), as well as increased concentrations of circulating glucocorticoids at the onset of inflammation downregulating inflammatory responses, is proof that glucocorticoids are modulators of inflammatory responses (243, 277). Adrenalectomized animals also showed greater microvascular responses to inflammatory mediators and cell migration to inflamed regions (243, 278). Glucocorticoids have powerful anti-inflammatory and immunosuppressive effects, such as lowering cytokine production or activity, reducing microvascular responses to inflammatory mediators, preventing leukocyte accumulation at inflamed sites, impeding phagocytic functions and microbicidal capacity of polymorphonuclear leukocytes, preventing the recruitment of mononuclear phagocytes to injured areas, and interfering with immune function (243, 278–280).

#### 6.6.5 Thyroid hormones

Thyroid hormones are associated with the oxidative and antioxidative status of an organism because of their role in regulating oxidative metabolism and in the formation of ROS (34, 39, 50–55, 281). Rats with thyroid dysfunction, produced by hormone injection or thyroidectomy, were evaluated for their ability to respond to noxious stimuli in an effort to determine the processes through which thyroid gland activity can influence the development of inflammatory reactions (243, 282, 283). Rats with hypothyroidism exhibit typical inflammatory responses, whereas animals with a sustained excess of circulating thyroid hormones exhibit consistently suppressed inflammatory responses (282, 283).

#### 6.6.6 Corticotropin releasing hormone

A study was conducted to investigate the impact of administering Corticotropin releasing hormone (CRH) to induce psychosocial stress in DSS-treated colitic mice, specifically by examining the potential enhancement of autophagy in intestinal macrophages. The inflammatory challenges associated with IBD led to increased autophagy in both intestinal macrophages and murine bone marrow-derived macrophages, and these effects were further increased by CRH (284).

#### 6.6.7 Adipokines

IBD is distinguished by symptoms such as reduced appetite, inadequate nutrition, changes in body composition, and the enlargement of mesenteric white adipose tissue (mWAT) (285, 286). Adipokines, namely leptin, adiponectin, and resistin, play a significant role in anorexia, malnutrition, changes in body composition, and hypertrophy of mWAT (285, 286). Studies have demonstrated that there is an increased expression of leptin, adiponectin, and resistin in mWAT in individuals diagnosed with CD.

##### 6.6.7.1 Leptin

Leptin serves as a regulator of diverse immune and inflammatory reactions in addition to its metabolic and endocrine roles. Leptin has the ability to initiate activation and alter the pattern of cytokine production, favoring a Th1 response by promoting the release of IL-2 and IFN-γ while inhibiting the secretion of IL-4 (287). Additionally, it directly stimulates the expression and release of IL-1a, IL-1b, IL-6, and TNFα by T-cells (288). Leptin was found to be expressed and released into the intestinal lumen by inflamed colonic epithelial cells in patients diagnosed with UC. Leptin, in turn, elicited damage to the epithelial wall and infiltration of neutrophils (289). There was a higher level of leptin mRNA expression in the mesenteric white adipose tissue (mWAT) of patients with IBD compared to the control group suggesting that leptin may play a role in the inflammatory process by increasing the expression of mesenteric TNFα (290). The study found that individuals with IBD had lower levels of serum leptin compared to a control group of healthy individuals. This difference was observed regardless of factors such as sex, age, CRP levels, years since diagnosis, and disease activity and localization. The study found that individuals with a BMI greater than or equal to 25 exhibited significantly lower levels of serum leptin compared to individuals with a BMI below 25 (286). The administration of infliximab, an anti-TNFα biologic agent, to individuals diagnosed with CD resulted in an elevation in leptinaemia levels. This suggests that TNFα plays a significant role in suppressing the production of leptin in patients with CD (291).

##### 6.6.7.2 Adiponectin

Adiponectin production and TNFα are mutually suppressed, and their actions are antagonistic in the target tissues (292). Additionally, *in vitro* studies have shown that IL-6 reduces adiponectin levels (292). The induction of adiponectin resulted in the production of IL-10 and interleukin-1 receptor antagonist (IL-1Ra) in human peripheral blood mononuclear cells (PBMC), macrophages, and dendritic cells (DC). Additionally, it hindered the production of IFN-γ in macrophages. The macrophages treated with adiponectin demonstrated a decreased ability to engulf particles and a diminished immune response to cells from a different individual (293). Previous studies have suggested a potential protective role of adiponectin against OS (294). Conversely, it has been observed that OS leads to a reduction in adiponectin secretion in 3T3-L1 adipocytes (295). Adiponectin can inhibit the enhanced cytotoxic activity of natural killer (NK) cells that are induced by IL-2, as well as the production of IFN-γ (296). Studies conducted using the adiponectin knock-out (KO) murine model revealed that adiponectin KO mice exhibited a notably more severe form of colitis in comparison to their wild-type counterparts. However, the severity of colitis was significantly reduced when adiponectin was supplemented. Additionally, it was observed that adiponectin exhibited inhibitory effects on the production of IL-8 in HT-29 cells stimulated with LPS, suggesting that adiponectin may possess a direct anti-inflammatory impact on intestinal epithelial cells (297). However, other studies showed contradictory results on the effect of adiponectin on the development of colitis and restoration of inflammation (298). There is an elevated secretion of adiponectin from mWAT in patients with CD who have undergone surgery, in comparison to patients with diverticulitis and colon carcinoma. Also, serum adiponectin levels are increased in patients with IBD versus healthy controls (286).

##### 6.6.7.3 Resistin

The secretion of resistin from mWAT in patients who underwent surgery for colon cancer or diverticulitis was observed to be significantly lower when compared to the secretion from the adipose tissue adjacent to the affected intestine in patients with CD (285). It was noted that the administration of steroids led to a reduction in resistin production in CD patients (299). The levels of serum resistin in patients with IBD (with active disease and also with quiescent disease) are elevated in comparison to healthy controls.

##### 6.6.7.4 Ghrelin

In chronic DSS-induced colitis in ghrelin KO mice, due to the absence of endogenous ghrelin, the DAI was reduced, and the infiltration of neutrophils was delayed compared to wild-type (300). The introduction of ghrelin either at the onset of the disease or a few days after colitis had developed resulted in ameliorating both the clinical and histopathologic severity of the disease. This therapeutic effect was observed alongside the suppression of inflammatory and Th1-driven autoimmune response, as well as elevated levels of IL-10 (301). Ghrelin mRNA expression and its receptor were found to be increased in TNBS-induced colitic murine models. Serum ghrelin levels were increased in patients with IBD, regardless of whether the disease was active or in remission and were higher in male versus female patients. Ghrelin levels were higher in patients with ileal CD compared to those with colonic CD. In contrast, Peracchi et al. (302) demonstrated that individuals with active IBD exhibited elevated levels of circulating ghrelin in comparison to both healthy controls and patients in a state of remission.

#### 6.6.8 Type 2 diabetes and IBD

Previous research conducted on animals has indicated that the presence of peroxisome proliferator-activated receptor γ (PPAR γ) in the intestinal epithelial cells, macrophages, and T cells of mice with experimental IBD demonstrates immunoregulatory effects and potentially plays a role in the prevention of intestinal inflammation (303, 304). The administration of the gamma subtype of peroxisome proliferator-activated receptors (PPARγ) ligands has been demonstrated to reduce the production of inflammatory cytokines, such as IL-1β and TNF-α, as well as inhibit the proliferation of inflammatory cells and the expression of specific adhesion molecules (305). The thiazolidinedione (TZD) antidiabetic medications for the treatment of type 2 diabetes function as ligands for the gamma subtype of PPARs. Several trials with TZD in the context of IBD have yielded intriguing outcomes (237, 306, 307). In a multicenter randomized, double-blind, placebo-controlled clinical trial, the effectiveness of rosiglitazone in managing UC with mild to moderate activity has been demonstrated (306). Here, 17% of patients who received treatment with rosiglitazone were able to achieve remission. Significant clinical improvement was observed in as short as 4 weeks, with enhanced quality of life by the 8th week (306). In another study, it was determined that TZD does not confer any discernible benefits in comparison to alternative oral antidiabetic drugs with regard to the prevention of UC-related flares. Nevertheless, the utilization of TZD may diminish the probability of experiencing more severe disease flares that necessitate oral steroid treatment (307).

#### 6.6.9 Melatonin

Melatonin (N-acetyl-5-methoxytryptamine) has several unique scavenging effects making it an exceptionally potent direct free radical scavenger and multifunctional antioxidant (34, 308, 309). Melatonin is an electron-rich molecule that can react with free radicals to produce stable compounds that are excreted in the urine (310). Melatonin is frequently referred to as a suicidal or terminal antioxidant because it eliminates free electrons from the system throughout its chemical rearrangement, and each of the rearrangement products is again a potent antioxidant. Melatonin acts as an indirect antioxidant, increasing AOEs, including SOD, CAT, GPx, GR (311, 312), and glucose-6-phosphate dehydrogenase (G6PD) (313). Increased intracellular levels of the antioxidant GSH are generated as a consequence of melatonin stimulating γ-glutamyl cysteine synthetase (the rate-limiting enzyme in GSH formation) and GR (the enzyme converting GSSG to GSH) (314, 315). Melatonin also potentiates the function of the mitochondrial electron transport chain, which reduces free radical generation and electron leakage (316). Melatonin inhibits the production of free radicals by acting as a negative modulator of several pro-oxidant enzymes including 5-lipoxygenase, 12-lipoxygenase, and NO synthase (309). It also prevents mitochondrial damage and reduces NF-κB signaling, suggesting a repair mechanism for intestinal injury caused by OS (317). Decreased levels of hydroxyl radical (HO•), peroxynitrite (ONOO^-^), RO_2_^•^, and singlet oxygen, and decreased expression of COX-2 and iNOS and NF-kB activation are all antioxidant effects of melatonin (318, 319), pointing to the possibility that melatonin is beneficial in UC by lowering inflammation and regulating OS. Pineal-derived melatonin and *de novo* synthesis in the GI tract both produce melatonin which helps regulate gut immunity and intestinal barrier integrity. Due to its antiapoptotic action and ability to decrease bacterial translocation across epithelia, melatonin can limit mucosal damage (73). Animal studies have demonstrated that melatonin treatment reduces inflammation by blocking the production of IL-10, IFN-γ, TNF-α, IL-6, and NO^•^ (320) (**Supplementary Table 1**). In murine colitis, melatonin reduces the generation of ROS and reactive nitrogen species (RNS), characterized by lowering colonic MDA levels and MPO activity and improved antioxidant defenses with increased GSH and SOD levels in the colon (134).

By modulating autophagy and Nrf2 signaling pathways, melatonin slows the progression of colitis-associated colon cancer in mice. In this colon cancer model, melatonin decreased the levels of inflammatory markers IL-6, IL-17, TNF-α, NF-κB, COX-2 and STAT3, as well as reduced DNA damage and OS indicated by reduced TBARS and increased GSH levels (**Supplementary Table 1**). The decrease in the expression of Beclin-1 and the LC3-II/LC3-I ratio, along with an increased expression of p62, indicate melatonin-inhibited cancer-associated autophagy. These results are like those showing upregulation of Nrf2 and AOEs NQO-1 and HO-1 in melatonin-treated mice with colon cancer (321). Another study investigated the role of oral melatonin therapy on OS in dogs before and after ovariohysterectomy (OHE) (135). The levels of SOD, GPx, and CAT were increased, and MDA decreased in ovariohysterectomized dogs (**Supplementary Table 1**) that received melatonin compared to those of the control group. These preliminary findings suggest that melatonin administration may reduce OS induced by OHE in dogs. Adjuvant melatonin treatment may aid in maintaining remission in patients with UC (136). In this clinical trial, patients receiving melatonin adjuvant treatment-maintained remission (e.g., lower disease activity scores and inflammatory biomarkers such as CRP levels with increased hemoglobin concentrations) over 12 months of observation (136).

## 7 Conclusion

Oxidative stress plays a crucial role in the pathogenesis of IBD and is closely associated with the development of intestinal inflammation in humans and animals. Therapeutic strategies involving natural antioxidant products have shown benefit in numerous human studies and animal models. An improved comprehension of the free radical biology mediating GI disease is essential for developing effective future treatments to reduce intestinal inflammation and improve the quality of life in affected individuals. Also, hormonal intervention holds potential importance in the context of IBD and cannot be disregarded. In this review, we have discussed the major antioxidant and anti-inflammatory properties of various phytochemicals and hormones in IBD. We have also discussed significant evidence-based observations, including the results from clinical trials using natural antioxidants or their modified formulations. Based on the existing scientific evidence, it appears likely that future therapies will include antioxidants with standard treatments or even as an alternative medical option in humans and animals with IBD.

## Author contributions

Writing—original draft preparation, revisions, figures, review, and editing DKS, Writing—review, and editing RMH, BP, AP, VKY, DW, AEJ. All authors contributed to the article and approved the submitted version.
