# Supplementary material for: Oxidative stress, hormones, and effects of natural antioxidants on intestinal inflammation in inflammatory bowel disease
Source: Front Endocrinol (Lausanne). 2023 Aug 28;14:1217165. doi: 10.3389/fendo.2023.1217165 (PMC10493311; doi:10.3389/fendo.2023.1217165)
Supplement: Supplementary file 1 [file Table_1.docx]

**Supplementary Table-1:** Polyphenolic compounds and hormones targeting cellular signaling pathways as potential IBD treatments.

|  | **Phytochemicals/hormones*** | **Protective Mechanism** | **Antioxidant Effects (ROS/RNS Scavenging activity)** | **Anti-inflammatory Effects** | **Models** |
| --- | --- | --- | --- | --- | --- |
| Polyphenolic substances | Resveratrol | - Inhibition of inflammatory transcription factors - Inhibition of pro-inflammatory cytokines - Modulating AOEs - ROS/RNS scavenging or reducing OS parameters | MDA ↓ | MPO↓ | - In vitro studies using murine RAW 264.7 macrophages (90,91) - TNBS-induced colitis murine models (91) - Patients with UC (92) |
|  |  |  | GPX↑ | IL-1↓ |  |
|  |  |  |  | IL-6↓ |  |
|  |  |  |  | TNF-α↓ |  |
|  |  |  |  | iNOS↓ |  |
|  |  |  |  | NO↓ |  |
|  |  |  |  | COX↓ |  |
|  |  |  |  | SIRT1↑ |  |
|  |  |  |  | NF-κB↓ |  |
| Polyphenolic substances | Curcumin | - Inhibition of inflammatory transcription factors - Inhibition of pro-inflammatory cytokines - Imbalance in the level of immune cells - AMPK pathway and autophagy - Modulating small antioxidant molecules - Modulating AOEs - ROS/RNS scavenging or reducing OS parameters | MDA ↓ | IL-1↓ | - In vitro studies using rat vascular smooth muscle cells (93) - TNBS-induced colitis murine models (94) - DSS-induced colitis guinea pigs (95) - Patients with UC (92) |
|  |  |  | PC ↓ | IL-1β↓ |  |
|  |  |  | MPO↓ | IL-6↓ |  |
|  |  |  | GST↑ | IL-8↓ |  |
|  |  |  | GSH↑ | IL-12↓ |  |
|  |  |  |  | IL-27↓ |  |
|  |  |  |  | MCP-1↓ |  |
|  |  |  |  | TLR4↓ |  |
|  |  |  |  | MyD88↓ |  |
|  |  |  |  | NF-κB↓ |  |
|  |  |  |  | NO↓ |  |
| Polyphenolic substances | Quercetin | - Inhibition of inflammatory transcription factors - Inhibition of pro-inflammatory cytokines - Activation of anti-inflammatory cytokines - Restoring Tight junction proteins, intestinal barriers and epithelial structure - Modulating small antioxidant molecules - ROS/RNS scavenging or reducing OS parameters | LPx↓ | NF-κB↓ | - Murine colitis-derived intestinal organoids (96) - DSS-colitis murine model (88) |
|  |  |  | MPO↓ | NO↓ |  |
|  |  |  | GSH↑ | COX-2↓ |  |
|  |  |  |  | PGE2↓ |  |
|  |  |  |  | iNOS↓ |  |
|  |  |  |  | TNF-α↓ |  |
|  |  |  |  | IL-1β↓ |  |
|  |  |  |  | IL-6↓ |  |
|  |  |  |  | IL-33↓ |  |
|  |  |  |  | AP-1↓ |  |
|  |  |  |  | IL-10↑ |  |
| Polyphenolic substances | Green tea flavonoids | - Inhibition of pro-inflammatory cytokines - Modulating AOEs - ROS/RNS scavenging or reducing OS parameters | MDA ↓ | TNF-α↓ | - In vitro studies using murine RAW 264.7 macrophages (97) - DSS-murine model of colitis (87) |
|  |  |  | MPO↓ | PGE2↓ |  |
|  |  |  | SOD↑ | iNOS↓ |  |
|  |  |  | GPx↑ | COX-2↓ |  |
|  |  |  | NO↓ | MyD88↓ |  |
|  |  |  |  | TRIF↓ |  |
| Polyphenolic substances | Caffeic acid phenethyl ester | - Inhibition of inflammatory transcription factors - Inhibition of pro-inflammatory cytokines - Restoring Tight junction proteins, intestinal barriers and epithelial structure - Nrf2 cytoprotective pathway - Modulating AOEs - ROS/RNS scavenging or reducing OS parameters | ROS↓ | IL-1↓ | - In vitro studies using RAW 264.7 cells (98) - DSS- murine model (99) |
|  |  |  | MDA↓ | IL-6↓ |  |
|  |  |  | Nrf2↑ | IL-8↓ |  |
|  |  |  | HO-1↑ | IL-12↓ |  |
|  |  |  | NQO-1↑ | iNOS↓ |  |
|  |  |  |  | COX-2↓ |  |
|  |  |  |  | TLR4↓ |  |
|  |  |  |  | MyD88↓ |  |
|  |  |  |  | NF-κB↓ |  |
|  |  |  |  | AP-1↓ |  |
|  |  |  |  | TNF-α↓ |  |
|  |  |  |  | Phosphorylation of PI3K↓ |  |
|  |  |  |  | Phosphorylation of Akt↓ |  |
| Polyphenolic substances | Luteolin | - Inhibition of inflammatory transcription factors - Inhibition of pro-inflammatory cytokines - Modulating AOEs - ROS/RNS scavenging or reducing OS parameters | MDA↓ | NF-κB↓ | - LPS-induced acute lung injury (ALI) murine model (100) |
|  |  |  | SOD↑ | TNF-α↓ |  |
|  |  |  | CAT↑ | ICAM-1↓ |  |
|  |  |  |  | TBK1-kinase activity↓ |  |
|  |  |  |  | IL-6↓ |  |
|  |  |  |  | IL-12↓ |  |
|  |  |  |  | IL-27↓ |  |
|  |  |  |  | IP-10↓ |  |
|  |  |  |  | IFN-β↓ |  |
|  |  |  |  | CXCL9↓ |  |
| Polyphenolic substances | Xanthohumol | - Inhibition of inflammatory transcription factors - Inhibition of pro-inflammatory cytokines - Restoring Tight junction proteins, intestinal barriers and epithelial structure - ROS/RNS scavenging or reducing OS parameters | MDA↓ | COX-2↓ | - In vitro studies using rat small intestine epithelial IEC-6 cell line (101) - DSS-treated mice (101) |
|  |  |  | NO↓ | iNOS↓ |  |
|  |  |  |  | IFN-γ↓ |  |
|  |  |  |  | NF-κB↓ |  |
|  |  |  |  | NF-κB p65, p50 and p105 nuclear translocation↓ |  |
|  |  |  |  | *A1α*↓ |  |
|  |  |  |  | *A20*↓ |  |
|  |  |  |  | *Bcl-xL*↓ |  |
|  |  |  |  | *c-myc*↓ |  |
| Polyphenolic substances | Genistein | - Inhibition of inflammatory transcription factors - Inhibition of pro-inflammatory cytokines - Modulating AOEs | SOD↑ | IL-1α↓ | - In vitro studies using BV2 mouse microglial cell line (102), RAW264.7 macrophages (103), human umbilical vein endothelial cells (104) - DSS murine colitis model (105) - Obese postmenopausal women (106) |
|  |  |  | CAT↑ | IL-1β↓ |  |
|  |  |  | GPx↑ | IL-6↓ |  |
|  |  |  | GR↑ | PGE2↓ |  |
|  |  |  | GST↑ | TNF-α↓ |  |
|  |  |  | NQO-1↑ | NF-κB↓ |  |
|  |  |  | NO↓ | TLR4↓ |  |
|  |  |  |  | MyD88↓ |  |
|  |  |  |  | IFN-β↓ |  |
|  |  |  |  | CSF-2↓ |  |
|  |  |  |  | CSF-3↓ |  |
|  |  |  |  | CCL2↓ |  |
|  |  |  |  | CXCL10↓ |  |
|  |  |  |  | IκBα↓ |  |
|  |  |  |  | COX-2↓ |  |
|  |  |  |  | VCAM-1↓ |  |
| Polyphenolic substances | Alpinetin | - Inhibition of inflammatory transcription factors - Inhibition of pro-inflammatory cytokines - Restoring Tight junction proteins, intestinal barriers and epithelial structure - Nrf2 cytoprotective pathway - Modulating AOEs - ROS/RNS scavenging or reducing OS parameters | MPO↓ | DAI↓ | - LPS-treated RAW 264.7 macrophages (107) - DSS-treated mice (108) - LPS-induced acute lung injury murine model (107) |
|  |  |  | SOD↑ | TLR4↓ |  |
|  |  |  | MDA↓ | IκBα↓ |  |
|  |  |  | Nrf2↑ | NF-κB↓ |  |
|  |  |  | HO-1↑ | IL-1β↓ |  |
|  |  |  |  | IL-6↓ |  |
|  |  |  |  | TNF-α↓ |  |
| Polyphenolic substances | Proanthocyanidins | - Inhibition of inflammatory transcription factors - Modulating small antioxidant molecules - Modulating AOEs - ROS/RNS scavenging or reducing OS parameters | SOD↑ | NF-κB activation↓ | - TNBS-induced colitis in rats (109) |
|  |  |  | GPx↑ | Iκκ activation↓ |  |
|  |  |  | GSH↑ | COX-2↓ |  |
|  |  |  | MPO↓ |  |  |
|  |  |  | ROS↓ |  |  |
|  |  |  | MDA↓ |  |  |
|  |  |  | NO↓ |  |  |
|  |  |  | iNOS↓ |  |  |
| Polyphenolic substances | Anthocyanins | - Inhibition of inflammatory transcription factors - Inhibition of pro-inflammatory cytokines - Signal transducer activator of transcription - Imbalance in the level of immune cells - Nrf2 cytoprotective pathway - Modulating AOEs - ROS/RNS scavenging or reducing OS parameters | CAT↑ | ↓IFN-γ activation of STAT1 and STAT3 | - Human monocytic THP-1 cells (110) - LPS/IFN-γ activated macrophages (111,112) - Murine DSS-colitis (86) - Patients with UC (92) |
|  |  |  | SOD↑ | ↓Activation of SAPK/JNK |  |
|  |  |  | GPx↑ | p38MAPK↓ |  |
|  |  |  | GR↑ | TNF-α↓ |  |
|  |  |  | MPO↓ | MCP-1↓ |  |
|  |  |  | Nrf2↑ | IL-1α↓ |  |
|  |  |  | 8-HdG↓ | IL-1β↓ |  |
|  |  |  |  | IL-6↓ |  |
|  |  |  |  | IL-17↓ |  |
|  |  |  |  | ICAM-1↓ |  |
|  |  |  |  | iNOS↓ |  |
|  |  |  |  | COX-2↓ |  |
|  |  |  |  | NF-κB↓ |  |
|  |  |  |  | CRP↓ |  |
|  |  |  |  | Fecal calprotectin↓ |  |
| Polyphenolic substances | Silymarin | - Inhibition of inflammatory transcription factors - Inhibition of pro-inflammatory cytokines - Nrf2 cytoprotective pathway - Modulating small antioxidant molecules - Modulating AOEs - ROS/RNS scavenging or reducing OS parameters | Redox balance↑ | NF-κB↓ | - TNBS-induced colitic rats (113) - Patients with UC, pediatric CD patients (114) |
|  |  |  | AOE↑ | IL-1β↓ |  |
|  |  |  | Nrf2↑ | TNF-α↓ |  |
|  |  |  | TBARS↓ |  |  |
|  |  |  | MPO↓ |  |  |
|  |  |  |  |  |  |
| Phenolic substances | Thymol | - Inhibition of inflammatory transcription factors - Inhibition of pro-inflammatory cytokines - Restoring Tight junction proteins, intestinal barriers, and epithelial structure - ROS/RNS scavenging or reducing OS parameters | ROS↓ | NF-κB↓ | - In vitro studies using Caco-2 cells (115) and LPS induced RAW264.7 cells (116), J774.1 mouse macrophages (117), porcine IPEC-J2 monolayer cell model (118) - DSS-induced murine colitis model (119) - Acetic acid-induced colitis murine model (117,120) |
|  |  |  | MDA↓ | IL-1β↓ |  |
|  |  |  | MPO↓ | IL-6↓ |  |
|  |  |  | NO↓ | IL-8↓ |  |
|  |  |  |  | TNF-α↓ |  |
|  |  |  |  | TLR4↓ |  |
|  |  |  |  | NF-κB p65 nuclear translocation↓ |  |
|  |  |  |  | Activation of p-p38, p-JNK, and p-ERK↓ |  |
|  |  |  |  | COX-2↓ |  |
|  |  |  |  | MAPK↓ |  |
|  |  |  |  | AP-1↓ |  |
|  |  |  |  | STAT-3↓ |  |
| Alkaloids | Berberine | - Inhibition of inflammatory transcription factors - Inhibition of pro-inflammatory cytokines - Restoring Tight junction proteins, intestinal barriers and epithelial structure - AMPK pathway and autophagy - Modulating AOEs - ROS/RNS scavenging or reducing OS parameters | Superoxide ↓ | IL-1β↓ | - RAW264.7 macrophages (121) - DSS-treated mice (122) - A double-blind placebo-controlled phase I trial on UC patients (123) |
|  |  |  | MPO↓ | IL-6↓ |  |
|  |  |  | NOX↓ | IL-17↓ |  |
|  |  |  | SOD↑ | NF-κB↓ |  |
|  |  |  | gp91phox (a plasma membrane subunit of NADPH oxidase)↓ | IFN-γ↓ |  |
|  |  |  |  | TNF-α↓ |  |
|  |  |  |  | iNOS↓ |  |
|  |  |  |  | KC↓ |  |
|  |  |  |  | ICAM-1↓ |  |
| Storage polysaccharides | Tamarind xyloglucan | - Inhibition of pro-inflammatory cytokines - Activation of anti-inflammatory cytokines - Restoring Tight junction proteins, intestinal barriers and epithelial structure - Imbalance in the level of immune cells - ROS/RNS scavenging or reducing OS parameters | MDA↓ | COX-2↓ | - DSS-induced colitis in mice (124,125) |
|  |  |  | Superoxide ↓ | p47↓ |  |
|  |  |  | iNOS↓ | Total Inflammatory Index↓ |  |
|  |  |  | NOX↓ | Infiltration of inflammatory cells↓ |  |
|  |  |  |  | TNF-α↓ |  |
|  |  |  |  | IL-10↑ |  |
| Other phytochemicals | Sulforaphane | - Inhibition of inflammatory transcription factors - Inhibition of pro-inflammatory cytokines - Nrf2 cytoprotective pathway - Modulating small antioxidant molecules - Modulating AOEs - ROS/RNS scavenging or reducing OS parameters | MDA↓ | TNF-α↓ | - Rats with colitis (8) |
|  |  |  | NO↓ | IL-6↓ |  |
|  |  |  | GPx↑ | COX-2↓ |  |
|  |  |  | GSH↑ | NF-κB↓ |  |
|  |  |  | Nrf2↑ | TLR4↓ |  |
|  |  |  | HO-1↑ | MyD88↓ |  |
|  |  |  |  | IL-1R associated kinase-1↓ |  |
|  |  |  |  | IFN regulatory factor 3 activation↓ |  |
| Food/spices | Flaxseed oil (α-linolenic acid) | - Inhibition of pro-inflammatory cytokines - Restoring Tight junction proteins, intestinal barriers and epithelial structure - Modulating small antioxidant molecules - Modulating AOEs - ROS/RNS scavenging or reducing OS parameters | SOD↑ | TNF-α↓ | - Human Caco-2 cells (126) - Murine DSS-colitis model (127) |
|  |  |  | GSH↑ |  |  |
|  |  |  | MPO↓ |  |  |
|  |  |  | MDA↓ |  |  |
|  |  |  |  |  |  |
| Food/spices | Ginger | - Inhibition of inflammatory transcription factors - Inhibition of pro-inflammatory cytokines - Activation of anti-inflammatory cytokines - Imbalance in the level of immune cells - ROS/RNS scavenging or reducing OS parameters | ROS↓ | IL-1β↓ | - In vitro studies using murine RAW 264.7 macrophages (128), human HuH7 hepatocyte cells (129) and human C28I2 chondrocyte cells (130) - DSS-colitis murine model (131) - Randomized, placebo-controlled, clinical trial involving patients with UC (132) |
|  |  |  | MDA↓ | IL-6↓ |  |
|  |  |  |  | IL-8↓ |  |
|  |  |  |  | IL-17↓ |  |
|  |  |  |  | SAA1↓ |  |
|  |  |  |  | NF-κB/COX-2↓ |  |
|  |  |  |  | TNF-α↓ |  |
|  |  |  |  | PGE2↓ |  |
|  |  |  |  | iNOS↓ |  |
|  |  |  |  | PI3K/Akt↓ |  |
|  |  |  |  | MyD88↓ |  |
|  |  |  |  | IkB kinase↓ |  |
|  |  |  |  | Iκκβ↓ |  |
|  |  |  |  | IRF3↓ |  |
|  |  |  |  | IFN-β↓ |  |
|  |  |  |  | IFN-γ↓ |  |
|  |  |  |  | IP-10↓ |  |
|  |  |  |  | IL-10↑ |  |
|  |  |  |  | IL-22↑ |  |
|  |  |  |  | TGF-β↑ |  |
| Hormones | Enterohormones (GLP-2) | - Restoring Tight junction proteins, intestinal barriers and epithelial structure - Modulating small antioxidant molecules - Modulating AOEs | GSSG↓ | IL-1β↓ | - Chemically induced colitis and indomethacin-induced murine model (133) |
|  |  |  | SOD↑ | NF-κB↓ |  |
|  |  |  | GSH↑ | COX-2↓ |  |
| Hormones | Melatonin | - Inhibition of inflammatory transcription factors - Inhibition of pro-inflammatory cytokines - Activation of anti-inflammatory cytokines - Signal transducer activator of transcription - Imbalance in the level of immune cells - Nrf2 cytoprotective pathway - Modulating small antioxidant molecules - Modulating AOEs - ROS/RNS scavenging or reducing OS parameters | ROS↓ | IFN-γ↓ | - Murine colitis (134) - Ovariohysterectomized dogs (135) - Clinical trial on patients with UC (136) |
|  |  |  | RNS (NO)↓ | IL-6↓ |  |
|  |  |  | MDA↓ | IL-10↓ |  |
|  |  |  | MPO↓ | IL-17↓ |  |
|  |  |  | Nrf2↑ | TNF-α↓ |  |
|  |  |  | NQO-1↑ | NF-κB↓ |  |
|  |  |  | HO-1↑ | COX-2↓ |  |
|  |  |  | GSH↑ | STAT3↓ |  |
|  |  |  | SOD↑ | CRP↓ |  |
|  |  |  | CAT↑ |  |  |
|  |  |  | GPx↑ |  |  |

*See sections 4.1 to 4.6 for details.

↑: Increase; ↓: Decrease; antioxidant enzymes (AOEs); oxidative stress (OS); thiobarbituric acid reactive substances (TBARS); ulcerative colitis (UC); dextran sulfate sodium (DSS); trinitrobenzene sulphonic acid (TNBS); Nuclear factor kappa B (NF-κB); nuclear factor erythroid 2–related factor 2 (Nrf2); tumor necrosis factor-alpha (TNF-α); interleukin (IL); malondialdehyde (MDA); myeloperoxidase (MPO); mitogen-activated protein kinase (MAPK); p38 mitogen-activated protein kinases (p38MAPK); signal transducer and activator of transcription 1 (STAT1); signal transducer and activator of transcription 3 (STAT3); cyclooxygenase (COX); cyclooxygenase-2 (COX-2); NADPH oxidase (NOX); inducible nitric oxide synthase (iNOS); Toll-like receptor 4 (TLR4); interferon beta (IFN-β); interferon-gamma (IFN-γ); lipopolysaccharide (LPS); myeloid differentiation 88 (MyD88); silent mating type information regulation 2 homolog 1 (SIRT1); C-reactive protein (CRP); adenosine 5’-monophosphate activated protein kinase (AMPK); nitric oxide (NO); monocyte chemoattractant protein-1 (MCP-1); prostaglandin E2 (PGE2); activator protein (AP-1); Toll/IL-1R domain-containing adaptor-inducing IFN-β (TRIF); heme oxygenase-1 (HO-1); NAD(P)H:quinone oxidoreductase (NQO-1); phosphoinositide 3-kinase (PI3K); protein kinase B (PKB, or Akt); intercellular adhesion molecule 1 (ICAM-1/CD54); TANK-binding kinase1 (TBK1); C-X-C motif chemokine ligand 9 (CXCL9); C-X-C motif chemokine ligand 10 (CXCL10); colony-stimulating factor 2 (CSF-2); colony-stimulating factor 3 (CSF-3); chemokine ligand 2 (CCL2); nuclear factor of kappa light polypeptide gene enhancer in B-cells inhibitor, alpha (IκBα); vascular adhesion molecule-1 (VCAM-1); disease activity index (DAI); IκB kinase (Iκκ); stress-activated protein kinase/c-Jun NH(2)-terminal kinase (SAPK/JNK); 8-hydroxydeoxyguanosine (8-HdG); keratinocyte chemoattractant (KC or CXCL1); serum amyloid A1 (SAA1); interferon regulatory factor 3 (IRF3); transforming growth factor β (TGF-β); Superoxide dismutase (SOD); catalase (CAT); glutathione peroxidases (GPx); glutathione reductase (GR); glutathione S-transferases (GST); reduced glutathione (GSH); oxidized glutathione (GSSG); protein carbonyl (PC); lipid peroxidation (LPx); reactive oxygen species (ROS).

**References**

8. Alattar A, Alshaman R, Al-Gayyar MMH. Therapeutic effects of sulforaphane in ulcerative colitis: effect on antioxidant activity, mitochondrial biogenesis and DNA polymerization. *Redox Rep* (2022) 27:128. doi: 10.1080/13510002.2022.2092378

86. Boussenna A, Cholet J, Goncalves-Mendes N, Joubert-Zakeyh J, Fraisse D, Vasson MP, et al. Polyphenol-rich grape pomace extracts protect against dextran sulfate sodium-induced colitis in rats. *J Sci Food Agric* (2016) 96:1260–8. doi: 10.1002/JSFA.7214

87. Brückner M, Westphal S, Domschke W, Kucharzik T, Lügering A. Green tea polyphenol epigallocatechin-3-gallate shows therapeutic antioxidative effects in a murine model of colitis. *J Crohns Colitis* (2012) 6:226–35. doi: 10.1016/J.CROHNS.2011.08.012/2/6-2-FIG043.JPEG

88. Shigeshiro M, Tanabe S, Suzuki T. Dietary polyphenols modulate intestinal barrier defects and inflammation in a murine model of colitis. *J Funct Foods* (2013)5:949–55. doi: 10.1016/J.JFF.2013.02.008

90. Martinez J, Moreno JJ. Effect of resveratrol, a natural polyphenolic compound, on reactive oxygen species and prostaglandin production. Biochem Pharmacol (2000) 59:865–70. doi: 10.1016/S0006-2952(99)00380-9

91. Yildiz G, Yildiz Y, Ulutas PA, Yaylali A, Ural M. Resveratrol pretreatment ameliorates TNBS colitis in rats. Recent Pat Endocr Metab Immune Drug Discovery (2015) 9:134. doi: 10.2174/1872214809666150806105737

92. Dziąbowska-Grabias K, Sztanke M, Zając P, Celejewski M, Kurek K, Szkutnicki S, et al. Antioxidant therapy in inflammatory bowel diseases. Antioxidants (2021) 10:1–18. doi: 10.3390/ANTIOX10030412

93. Meng Z, Yan C, Deng Q, Gao DF, Niu XL. Curcumin inhibits LPS-induced inflammation in rat vascular smooth muscle cells in vitro via ROS-relative TLR4-MAPK/NF-kB pathways. Acta Pharmacol Sin (2013) 34:901. doi: 10.1038/APS.2013.24

94. Zeng Z, Zhan L, Liao H, Chen L, Lv X. Curcumin improves TNBS-induced colitis in rats by inhibiting IL-27 expression via the TLR4/NF-kB signaling pathway. Planta Med (2013) 79:102–9. doi: 10.1055/S-0032-1328057/BIB

95. Sharma M, Sharma S,Wadhwa J. Improved uptake and therapeutic intervention of curcumin via designing binary lipid nanoparticulate formulation for oral delivery in inflammatory bowel disorder. *Artif Cells Nanomed Biotechnol* (2019) 47:45–55. doi: 10.1080/21691401.2018.1543191

96. Stallhofer J, Friedrich M, Konrad-Zerna A, Wetzke M, Lohse P, Glas J, et al. Lipocalin-2 is a disease activity marker in inflammatory bowel disease regulated by IL-17A, IL-22, and TNF-a and modulated by IL23R genotype status. *Inflammation Bowel Dis* (2015) 21:2327–40. doi: 10.1097/MIB.0000000000000515

97. Zhong Y, Chiou YS, Pan MH, Shahidi F. Anti-inflammatory activity of lipophilic epigallocatechin gallate (EGCG) derivatives in LPS-stimulated murine macrophages. *Food Chem* (2012) 134:742–8. doi: 10.1016/J.FOODCHEM.2012.02.172

98. Khan MN, Lane ME, McCarron PA, Tambuwala MM. Caffeic acid phenethyl ester is protective in experimental ulcerative colitis via reduction in levels of proinflammatory mediators and enhancement of epithelial barrier function. *Inflammopharmacology* (2018) 26:561–9. doi: 10.1007/S10787-017-0364-X

99. Mei Y, Wang Z, Zhang Y, Wan T, Xue J, He W, et al. FA-97, a new synthetic caffeic acid phenethyl ester derivative, ameliorates DSS-induced colitis against oxidative stress by activating nrf2/HO-1 pathway. *Front Immunol* (2020) 10:2969/FULL. doi: 10.3389/FIMMU.2019.02969/FULL

100. Kuo MY, Liao MF, Chen FL, Li YC, Yang ML, Lin RH, et al. Luteolin attenuates the pulmonary inflammatory response involves abilities of antioxidation and inhibition of MAPK and NFkB pathways in mice with endotoxin-induced acute lung injury. *Food Chem Toxicol* (2011) 49:2660–6. doi: 10.1016/J.FCT.2011.07.012

101. Cho JM, Yun SM, Choi YH, Heo J, Kim NJ, Kim SH, et al. Xanthohumol prevents dextran sulfate sodium-induced colitis via inhibition of IKKb/NF-kB signaling in mice. *Oncotarget* (2018) 9:866. doi: 10.18632/ONCOTARGET.23183

102. Jeong JW, Lee HH, Han MH, Kim GY, Kim WJ, Choi YH. Anti-inflammatory effects of genistein via suppression of the toll-like receptor 4-mediated signaling pathway in lipopolysaccharide-stimulated BV2 microglia. *Chem Biol Interact* (2014)

212:30–9. doi: 10.1016/J.CBI.2014.01.012

103. Cui S, Bilitewski U. Effect of genistein on the TLR and MAPK transduction cascades in lipopolysaccharide-stimulated macrophages. *Chin UB-X bao yu fen zi M yi xue za zhi* (2014) 30(3):233-236.

104. Jia Z, Babu PVA, Si H, Nallasamy P, Zhu H, Zhen W, et al. Genistein inhibits TNF-α-induced endothelial inflammation through the protein kinase pathway A and improves vascular inflammation in C57BL/6 mice. *Int J Cardiol* (2013) 168:2637.

doi: 10.1016/J.IJCARD.2013.03.035

105. Chen Y, Le TH, Du Q, Zhao Z, Liu Y, Zou J, et al. Genistein protects against DSS-induced colitis by inhibiting NLRP3 inflammasome via TGR5-cAMP signaling. *Int Immunopharmacol* (2019) 71:144–54. doi: 10.1016/J.INTIMP.2019.01.021

106. Llaneza P, González C, Fernandez-Iñarrea J, Alonso A, Diaz F, Arnott I, et al. Soy isoflavones, diet and physical exercise modify serum cytokines in healthy obese postmenopausal women. *Phytomedicine* (2011) 18:245–50. doi: 10.1016/

J.PHYMED.2010.07.011

107. Huo M, Chen N, Chi G, Yuan X, Guan S, Li H, et al. Traditional medicine alpinetin inhibits the inflammatory response in Raw 264.7 cells and mouse models. *Int Immunopharmacol* (2012) 12:241–8. doi: 10.1016/J.INTIMP.2011.11.017

108. Tan Y, Zheng C. Effects of alpinetin on intestinal barrier function, inflammation and oxidative stress in dextran sulfate sodium-induced ulcerative colitis mice. *Am J Med Sci* (2018) 355:377–86. doi: 10.1016/J.AMJMS.2018.01.002

109. Wang YH, Yang XL, Wang L, Cui MX, Cai YQ, Li XL, et al. Effects of proanthocyanidins from grape seed on treatment of recurrent ulcerative colitis in rats. *Can J Physiol Pharmacol* (2010) 88:888–98. doi: 10.1139/Y10-071

110. Roth S, Spalinger MR, Müller I, Lang S, Rogler G, Scharl M. Bilberry-derived anthocyanins prevent IFN-g-induced pro-inflammatory signalling and cytokine secretion in human THP-1 monocytic cells. *Digestion* (2014) 90:179–89.

doi: 10.1159/000366055

111. Olejnik A, Kowalska K, Kidoń M, Czapski J, Rychlik J, Olkowicz M, et al. Purple carrot anthocyanins suppress lipopolysaccharide-induced inflammation in the co-culture of intestinal Caco-2 and macrophage RAW264.7 cells. *Food Funct* (2016) 7:557–64. doi: 10.1039/C5FO00890E

112. Li L, Wang L, Wu Z, Yao L, Wu Y, Huang L, et al. Anthocyanin-rich fractions from red raspberries attenuate inflammation in both RAW264.7 macrophages and a mouse model of colitis. *Sci Rep* (2014) 4:6234. doi: 10.1038/SREP06234

113. Esmaily H, Hosseini-Tabatabaei A, Rahimian R, Khorasani R, Baeeri M, Barazesh-Morgani A, et al. On the benefits of silymarin in murine colitis by improving balance of destructive cytokines and reduction of toxic stress in the bowel cells. *Cent Eur J Biol* (2009) 4:204–13. doi: 10.2478/S11535-008-0053-2/METRICS

114. Koláček M, Muchová J, Dvořáková M, Paduchová Z, Žitňanová I, Čierna I, et al. Effect of natural polyphenols (Pycnogenol) on oxidative stress markers in children suffering from Crohn’s disease–a pilot study. *Free Radic Res* (2013) 47:624–34.

doi: 10.3109/10715762.2013.807508

115. Putaala H, Nurminen P, Tiihonen K. Effects of cinnamaldehyde and thymol on cytotoxicity, tight junction barrier resistance, and cyclooxygenase-1 and -2 expression in Caco-2 cells. *J Anim Feed Sci* (2017) 26:274–84. doi: 10.22358/JAFS/77058/2017

116. Chen J, Li DL, Xie LN, Ma Y r, Wu PP, Li C, et al. Synergistic anti-inflammatory effects of silibinin and thymol combination on LPS-induced RAW264.7 cells by inhibition of NF-kB and MAPK activation. *Phytomedicine* (2020) 78:153309. doi:10.1016/J.PHYMED.2020.153309

117. Gholijani N, Gharagozloo M, Farjadian S, Amirghofran Z. Modulatory effects of thymol and carvacrol on inflammatory transcription factors in lipopolysaccharidetreated macrophages. *J Immunotoxicol* (2016) 13:157–64. doi: 10.3109/1547691X.2015.1029145

118. Omonijo FA, Liu S, Hui Q, Zhang H, Lahaye L, Bodin JC, et al. Thymol improves barrier function and attenuates inflammatory responses in porcine intestinal epithelial cells during lipopolysaccharide (LPS)-induced inflammation. *J Agric Food Chem* (2019) 67:615–24. doi: 10.1021/ACS.JAFC.8B05480/ASSET/IMAGES/LARGE/JF-2018-054805_0007.JPEG

119. Mueller K, Blum NM, Mueller AS. Examination of the anti-inflammatory, antioxidant, and xenobiotic-inducing potential of broccoli extract and various essential oils during a mild DSS-induced colitis in rats. *ISRN Gastroenterol* (2013) 2013:1–14.

doi: 10.1155/2013/710856

120. Khazdair MR, Ghorani V, Alavinezhad A, Boskabady MH. Pharmacological effects of Zataria multiflora Boiss L. and its constituents focus on their anti-inflammatory, antioxidant, and immunomodulatory effects. *Fundam Clin Pharmacol*

(2018) 32:26–50. doi: 10.1111/FCP.12331

121. Cheng WE, Ying Chang M, Wei JY, Chen YJ, Maa MC, Leu TH. Berberine reduces Toll-like receptor-mediated macrophage migration by suppression of Src enhancement. *Eur J Pharmacol* (2015) 757:1–10. doi: 10.1016/J.EJPHAR.2015.03.013

122. Yan F, Wang L, Shi Y, Cao H, Liu L, Kay Washington M, et al. Berberine promotes recovery of colitis and inhibits inflammatory responses in colonic macrophages and epithelial cells in DSS-treated mice. *Am J Physiol Gastrointest Liver Physiol* (2012) 302:G504. doi: 10.1152/AJPGI.00312.2011

123. Xu L, Zhang Y, Xue X, Liu J, Li ZS, Yang GY, et al. A phase I trial of berberine in Chinese with ulcerative colitis. *Cancer Prev Res* (2020) 13:117–26. doi: 10.1158/1940-6207.CAPR-19-0258/37877/AM/A-PHASE-I-TRIAL-OF-BERBERINE-IN-CHINESEWITH

124. Ross EA, Miller MH, Pacheco A, Willenberg AR, Tigno-Aranjuez JT, Crawford KE. Intrarectal xyloglucan administration reduces disease severity in the dextran sodium sulfate model of mouse colitis. *Clin Exp Gastroenterol* (2021) 14:429–39.

doi: 10.2147/CEG.S325945

125. Periasamy S, Lin CH, Nagarajan B, Sankaranarayanan NV, Desai UR, Liu MY. Tamarind xyloglucan attenuates dextran sodium sulfate induced ulcerative colitis: Role of antioxidation. *J Funct Foods* (2018) 42:327–38. doi: 10.1016/J.JFF.2018.01.014

126. Miyamoto J, Mizukure T, Park SB, Kishino S, Kimura I, HIrano K, et al. A gut microbial metabolite of linoleic acid, 10-hydroxy-cis-12-octadecenoic acid, ameliorates intestinal epithelial barrier impairment partially via GPR40-MEK-ERK pathway. *J Biol Chem* (2015) 290:2902. doi: 10.1074/JBC.M114.610733

127. Zhou Q, Ma L, Zhao W, Zhao W, Han X, Niu J, et al. Flaxseed oil alleviates dextran sulphate sodium-induced ulcerative colitis in rats. *J Funct Foods* (2020) 64:103602. doi: 10.1016/J.JFF.2019.103602

128. Pan MH, Hsieh MC, Hsu PC, Ho SY, Lai CS, Wu H, et al. 6-Shogaol suppressed lipopolysaccharide-induced up-expression of iNOS and COX-2 in murine macrophages. *Mol Nutr Food Res* (2008) 52:1467–77. doi: 10.1002/MNFR.200700515

129. Li XH, McGrath KCY, Tran VH, Li YM, Duke CC, Roufogalis BD, et al. Attenuation of proinflammatory responses by S -[6]-Gingerol via inhibition of ROS/NF-Kappa B/COX2 activation in HuH7 cells. *Evidence-Based Complementary Altern*

*Med* (2013) 2013:146142. doi: 10.1155/2013/146142

130. Hosseinzadeh A, Bahrampour Juybari K, Fatemi MJ, Kamarul T, Bagheri A, Tekiyehmaroof N, et al. Protective Effect of Ginger (Zingiber officinale Roscoe) Extract against Oxidative Stress and Mitochondrial Apoptosis Induced by Interleukin-1b in Cultured Chondrocytes. *Cells Tissues Organs* (2017) 204:241–50. doi: 10.1159/000479789

131. Guo S, Geng W, Chen S, Wang L, Rong X, Wang S, et al. Ginger alleviates DSS induced ulcerative colitis severity by improving the diversity and function of gut microbiota. *Front Pharmacol* (2021) 12:632569. doi: 10.3389/FPHAR.2021.632569

132. Nikkhah-Bodaghi M, Maleki I, Agah S, Hekmatdoost A. Zingiber officinale and oxidative stress in patients with ulcerative colitis: A randomized, placebo-controlled, clinical trial. *Complement Ther Med* (2019) 43:1–6. doi: 10.1016/J.CTIM.2018.12.021

133. Skarbaliene J, Mathiesen JM, Larsen BD, Thorkildsen C, Petersen YM. Glepaglutide, a novel glucagon-like peptide-2 agonist, has anti-inflammatory and mucosal regenerative effects in an experimental model of inflammatory bowel disease in rats. *BMC Gastroenterol* (2023) 23:1–9. doi: 10.1186/S12876-023-02716-4/FIGURES/4

134. Tahan G, Gramignoli R, Marongiu F, Aktolga S, Cetinkaya A, Tahan V, et al. Melatonin expresses powerful anti-inflammatory and antioxidant activities resulting in complete improvement of acetic-acid-induced colitis in rats. *Dig Dis Sci* (2011) 56:715–20. doi: 10.1007/S10620-010-1364-5/FIGURES/4

135. Salavati S, Mogheiseh A, Nazifi S, Amiri A, Nikahval B. The effects of melatonin treatment on oxidative stress induced by ovariohysterectomy in dogs. *BMC Vet Res* (2021) 17:1–8. doi: 10.1186/S12917-021-02882-1/FIGURES/3

136. Wisniewska-Jarosinska M, Walecka-Kapica E, Jaworek J. Evaluation of melatonin effectiveness in the adjuvant treatment of ulcerative colitis. *J Physiol Pharmacol* (2011) 62(3):327-334.
